# Time-evolving self-assembly of a single-component fluorophore enables dynamic emission for temporal information encryption and self-erasing writing†

**DOI:** 10.1039/d6sc04670c

**Published:** 2026-08-25

**Authors:** Qiaona Zhang, Qing Liu, Leyong Wang, Tangxin Xiao

**Affiliations:** a School of Petrochemical Engineering, Changzhou University Changzhou 213164 China xiaotangxin@cczu.edu.cn; b School of Chemistry, Nanjing University Nanjing 210023 China

## Abstract

Time-evolving supramolecular systems capable of autonomously generating multiple emissive states represent a comparatively underexplored dimension for optical information processing, with their realization in single-component platforms featuring intrinsic dynamic controllability being particularly challenging. Herein, we report a tris(cyanostyryl)benzene derivative (TCSE) whose time-dependent self-assembly spontaneously produces distinct emissive states, enabling temporal information encryption within a single-component system. In THF/H_2_O solution, TCSE initially forms blue-emissive metastable nanoparticles, which gradually evolve into thermodynamically more stable green-emissive nanorods, generating a built-in time axis that can be directly visualized through dual-color fluorescence. In the solid state, TCSE exhibits reversible mechanofluorescence and enables self-erasing fluorescent writing. Distinct from conventional multicomponent or stimulus-responsive systems, this work establishes a single-component supramolecular platform that couples assembly kinetics with dynamic optical outputs, providing a new framework for temporal photonic information processing.

## Abstract 2

Leveraging intrinsic self-assembly, a single fluorophore transduces packing evolution into programmable temporally resolved emission, establishing a new paradigm for dynamic encryption and self-erasing optical materials.

## Graphical abstract

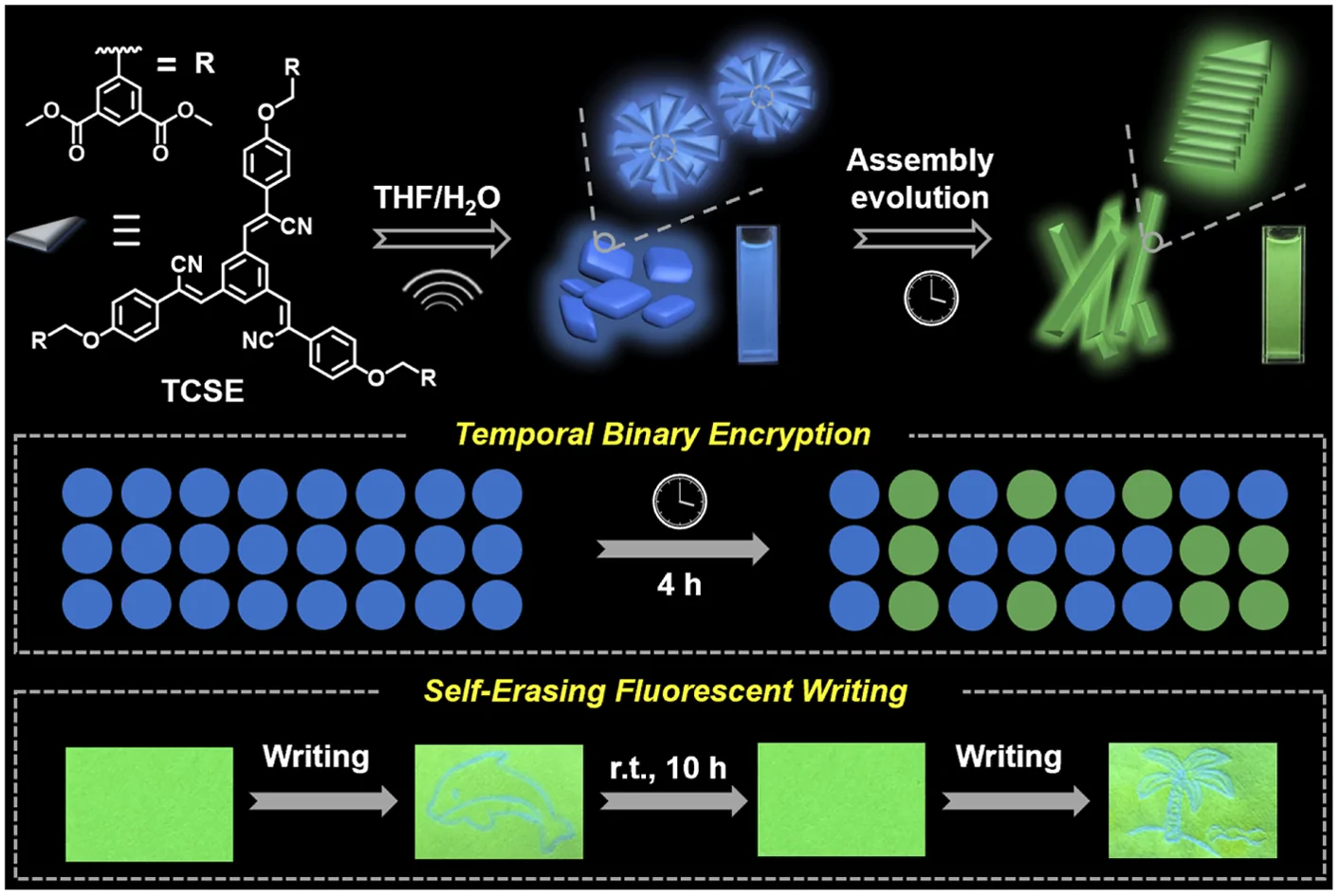

## Introduction

Time-resolved luminescent materials have recently attracted growing interest for advanced information encryption, as they enable dynamic encoding processes in which optical signals evolve over time.^1–8^ In contrast to static luminescent systems, the introduction of a temporal dimension allows information to appear, transform, or disappear within predefined time windows, thereby significantly enhancing security levels.^9–11^ However, achieving autonomous, multicolor, and precisely controllable temporal luminescence remains a fundamental challenge. Most reported systems rely on multicomponent assemblies, where time-dependent emission originates from processes such as energy transfer,^12–15^ host–guest complexation,^16–19^ metal–ligand coordination,^20–22^ or even fuel-driven dissipative assembly under nonequilibrium conditions.^23–25^ While effective, these strategies inherently suffer from structural complexity and sensitivity to component ratios, which can hinder precise control over their temporal luminescence behavior. In addition, multicomponent nature often gives rise to poorly defined kinetic pathways, compromising the predictability and programmability of their temporal evolution.

In contrast, single-component systems offer a conceptually simpler and potentially more robust platform for temporal information encoding.^26–29^ Nevertheless, current examples are scarce and predominantly limited to emission intensity variations rather than color evolution,^30^ or they require continuous external stimulation to drive dynamic processes.^31–33^ Meanwhile, precise regulation of temporal evolution kinetics, a key requirement for high-dimensional information encoding, remains largely unexplored in single-component systems. These limitations raise a fundamental question: can a single molecular system intrinsically encode temporal information through its own self-assembly pathway, enabling autonomous luminescence with multicolor evolution and precise kinetic control, without relying on multiple components or external inputs?

**Fig. 1:**
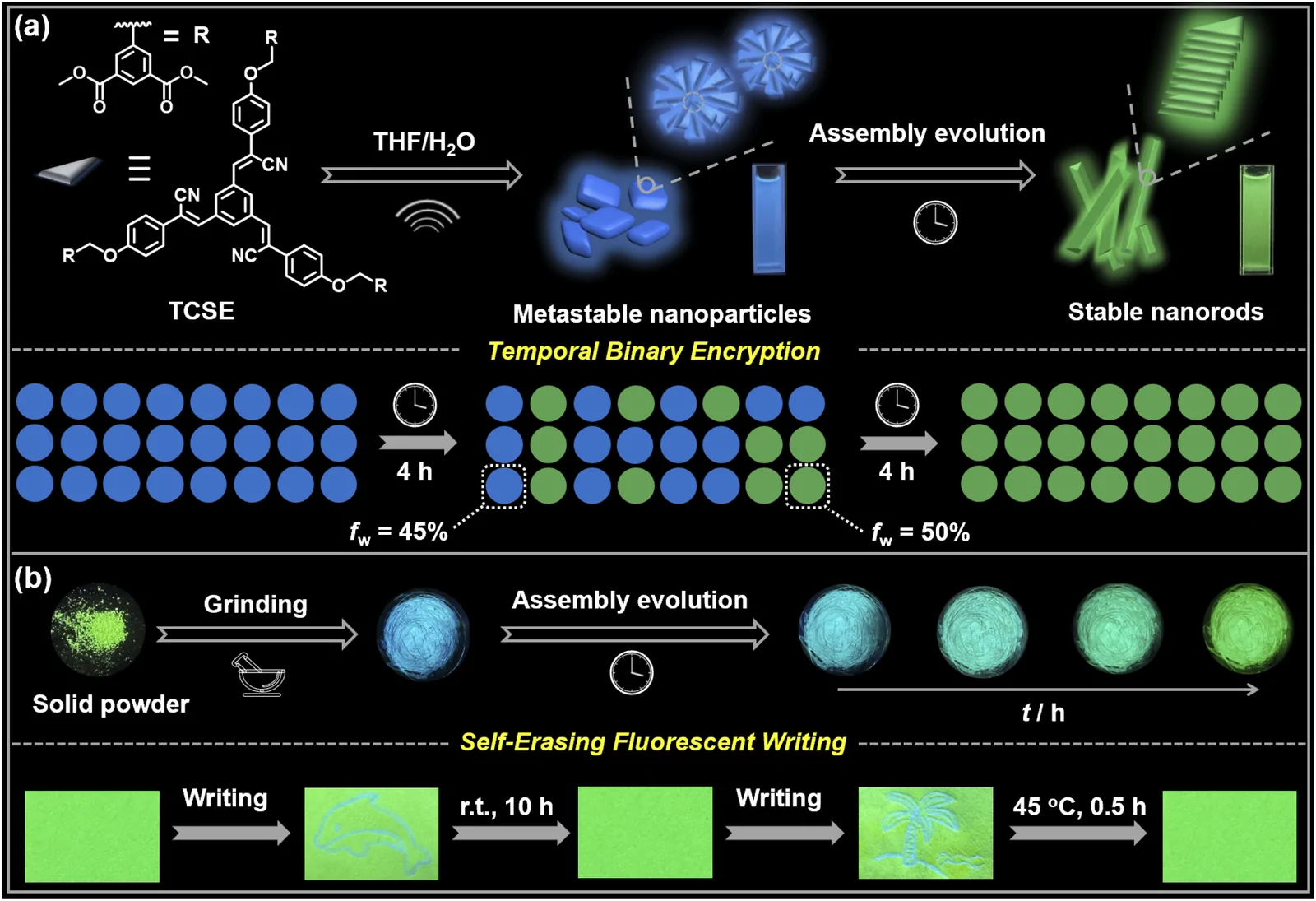
(a) Chemical structure of TCSE and schematic illustration of its time-dependent fluorescence color evolution in solution, together with its application in temporal binary information encryption. (b) Mechanofluorescence of TCSE powder and its application in self-erasing fluorescent writing.

Herein, we propose a single-component, self-assembly-driven strategy to achieve autonomous temporal luminescence with multicolor evolution and tunable kinetics. A tris(cyanostyryl)benzene derivative (TCSE) was rationally designed by integrating multiple aggregation-induced emission^34–38^ (AIE)-active cyanostyryl units into a single π-conjugated framework, enabling strong intermolecular interactions alongside restricted intramolecular motion (Fig. 1). This molecular design allows the system to undergo a progressive self-assembly pathway, during which distinct emissive states emerge intrinsically from evolving molecular packing rather than from energy transfer processes. In mixed solvent systems, TCSE spontaneously assembles into nanostructures accompanied by gradual emission color evolution from blue to green within a tunable time window, where the evolution rate can be readily modulated by adjusting the water fraction. In the solid state, TCSE further exhibits mechanochromic luminescence and self-erasable emission switching with controllable recovery kinetics. This work establishes a new paradigm for temporal information encoding governed by intrinsic self-assembly dynamics in single-component systems, offering a versatile platform for advanced anti-counterfeiting and intelligent optical materials.

## Results and discussion

Cyanostyryl (CS) groups are efficient AIE fluorophores and can also drive supramolecular self-assembly *via* π–π interactions.^39–43^ In our previous work, molecular systems incorporating one or two CS units were developed, demonstrating diverse functional applications.^44–47^ Building on these findings, we envisioned that integrating three CS groups within a single molecular framework would further enhance both the photophysical properties and the driving force for self-assembly. Guided by this rationale, we designed and synthesized TCSE, a tris(cyanostyryl)benzene (TCS) derivative bearing three dimethyl isophthalate substituents. The extended π-conjugated TCS core facilitates intermolecular interactions,^48,49^ while the bulky substituents enforce a nonplanar, multi-aryl conformation favorable for AIE. This combination of features renders TCSE a promising building block for supramolecular self-assembly in aqueous media, where cooperative π–π interactions, together with hydrophobic effects, are expected to drive the formation of ordered aggregates. The synthetic route to TCSE is provided in Scheme S1 (SI), and its structure was unambiguously confirmed by NMR spectroscopy and high-resolution ESI-MS (Fig. S1–S5, SI).

**Fig. 2:**
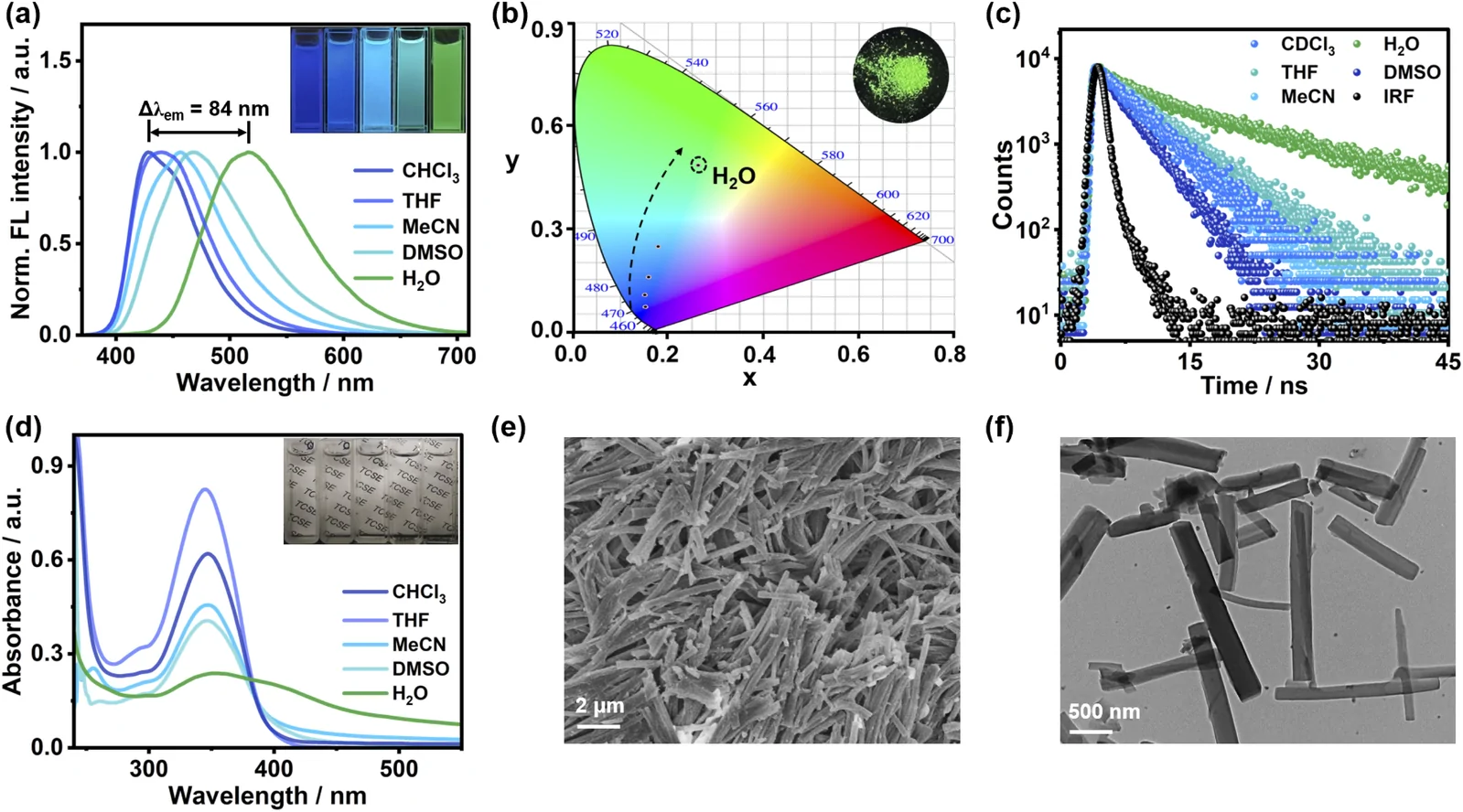
(a) Normalized fluorescence (FL) spectra of TCSE (4.0 × 10^−6^ M) in various solvents with different polarities. Inset: fluorescence images of TCSE in various solvents. (b) CIE 1931 chromaticity shifts of TCSE in various solvents. Inset: solid powder of TCSE. (c) Fluorescence decay profiles of TCSE in different solvents. (d) Absorption spectra of TCSE (1.0 × 10^−5^ M) in various solvents. Inset: images of TCSE in various solvents under ambient light. (e) SEM and (f) TEM images of TCSE nanorods.

The photophysical properties of TCSE was first investigated in various solvents. As shown in Fig. 2a, the normalized fluorescence (FL) spectra exhibit pronounced solvent-dependent behavior. With increasing solvent polarity from chloroform to DMSO and further to water, the emission undergoes a significant red shift of ∼84 nm, accompanied by a color change from blue to cyan to green (Fig. 2a, inset). The corresponding CIE 1931 chromaticity diagram shows only minor variations in organic solvents but a pronounced displacement in water (Fig. 2b), indicating the occurrence of π–π interaction-driven self-assembly probably occurred in aqueous media. Notably, the solid-state powder also exhibits intense green emission (Fig. 2b, inset), and the close similarity in emission color between the aqueous assemblies and the solid state suggests that the nanostructures formed in water adopt a packing motif resembling that of the bulk solid.

Time-resolved fluorescence measurements further revealed that TCSE exhibits its longest fluorescence lifetime in water (*τ* = 10.3 ns; Fig. 2c), together with an absolute fluorescence quantum yield of 18.83% (Fig. S6, SI). To gain further insight into this behavior, the radiative (*k*_r_) and nonradiative (*k*_nr_) decay rate constants were calculated from the measured fluorescence lifetimes and quantum yields (Table S1, SI). Upon self-assembly into nanorods, both *k*_r_ and *k*_nr_ decrease markedly compared with those in organic solvents, indicating that the ordered aggregates effectively suppress both radiative and nonradiative decay processes. Notably, the reduction in *k*_r_ is substantially greater than that in *k*_nr_, leading to a lower fluorescence quantum yield despite the prolonged fluorescence lifetime. This behavior can be attributed to strong intermolecular π–π interactions and excitonic coupling within the ordered aggregates.

Absorption spectra further support this aggregation behavior (Fig. 2d). In organic solvents, the absorption peak remains at ∼346 nm, whereas in water the spectrum broadens, the 346 nm peak decreases, and a shoulder appears at ∼410 nm, signaling aggregate formation. Under ambient light, the solutions show little visual change (Fig. 2d, inset), contrasting sharply with the fluorescence color variation under UV irradiation. Tyndall effect measurements confirm significant light scattering in water but negligible effects in organic solvents (Fig. S7, SI), corroborating the formation of unique supramolecular assemblies. Scanning electron microscopy (SEM) and transmission electron microscope (TEM) imaging of aqueous TCSE directly reveals uniform nanorods (Fig. 2e and f). Notably, the green emission forms immediately in water and remains stable over time, mirroring the solid-state color. These results demonstrate that TCSE strongly aggregates in water, with the nanorod structure representing a thermodynamically more stable state.

Notably, the pronounced bathochromic shift in fluorescence upon self-assembly in water is accompanied by only a slight red shift together with significant band broadening and tailing in the absorption spectra. Such spectral features are not characteristic of ideal H- or J-type aggregates, but instead suggest a non-ideal excitonic coupling arising from intermolecular π–π interactions within the assembled nanorods. To gain molecular-level insight into the relationship between intermolecular packing and the observed optical properties, density functional theory (DFT) calculations were subsequently performed. The minimum-energy geometries of the TCSE monomer, dimer, and trimer were optimized (Fig. S8, SI). Time-dependent DFT (TDDFT) calculations were then performed to correlate the aggregated structures with their corresponding spectral characteristics. The calculated S_0_–S_1_ energy gap at the optimized S_1_ geometries decreases from 3.69 to 3.16 eV upon aggregation (Table S2, SI), which is consistent with the experimentally observed red-shift of fluorescence emission. Independent gradient model based on Hirshfeld partition (IGMH)^50^ analysis reveals extensive intermolecular van der Waals interaction regions within both the dimer and trimer (Fig. S9, SI). These regions are dominated by pronounced π–π stacking interactions, which, together with the intrinsic π-conjugation of TCSE, promote the formation of ordered nanorod assemblies. Consistently, molecular orbital analysis reveals a progressive narrowing of the HOMO–LUMO gap upon going from the monomer to the trimer (Fig. S10, SI), accounting for the aggregation-induced red-shift of the absorption onset in aqueous media. Collectively, these results establish a direct correlation between supramolecular packing, electronic structure modulation, and the optical responses of TCSE in aqueous assemblies.

**Fig. 3:**
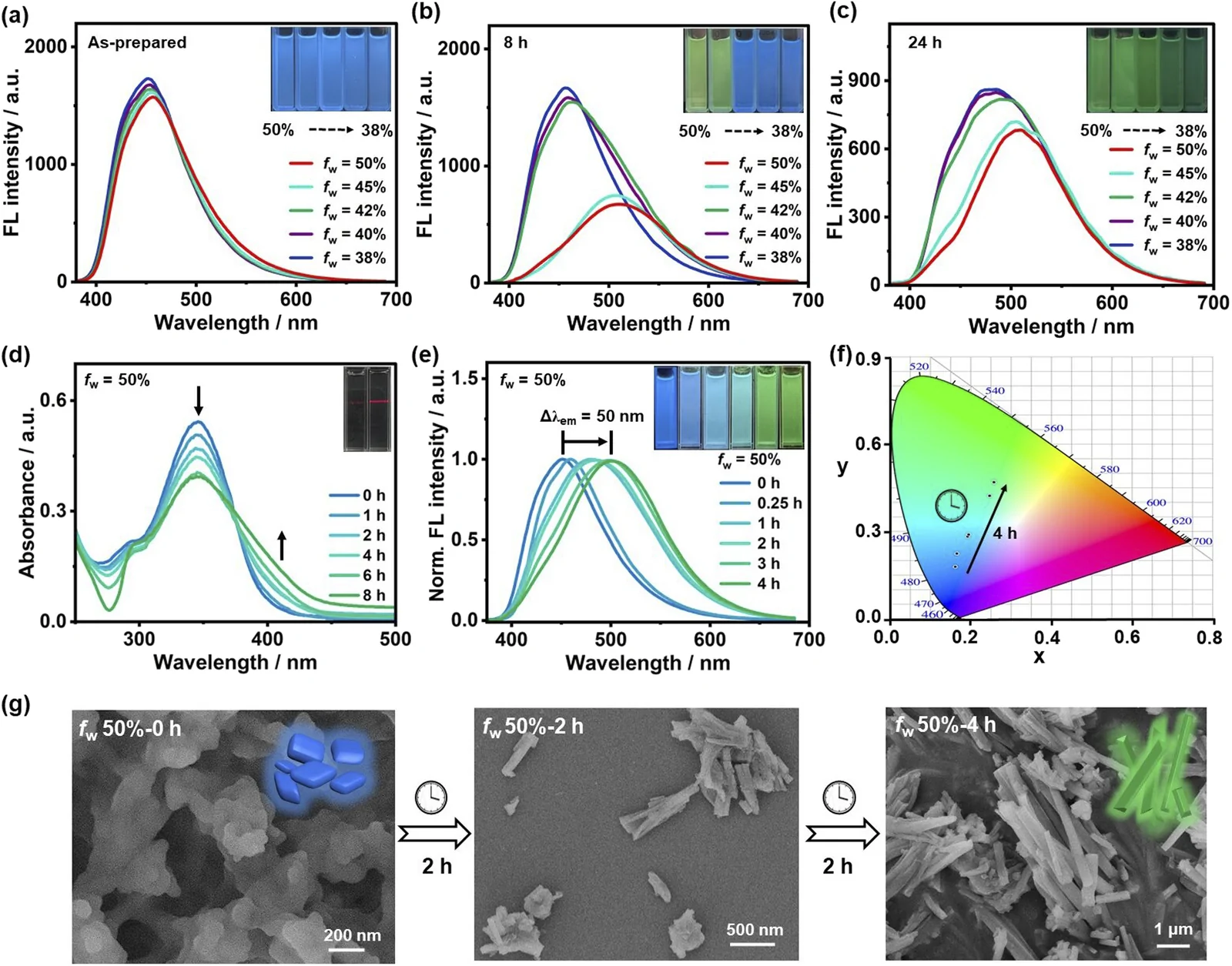
Fluorescence spectra of TCSE in THF/H_2_O mixtures at varying water fractions (*f*_w_ = 38–50%) for freshly prepared samples (a), after 8 h (b), and 24 h (c); insets show corresponding fluorescence photographs. (d) UV-vis absorption of TCSE (*f*_w_ = 50%) over time; inset: Tyndall effect at 0 h (left) and 4 h (right). (e) Normalized fluorescence spectra of TCSE (*f*_w_ = 50%) over time; inset: fluorescence images from 0 to 4 h. [TCSE] = 1.0 × 10^−5^ M, *λ*_ex_ = 365 nm. (f) CIE 1931 chromaticity shifts of TCSE (*f*_w_ = 50%) over time. (g) Time-dependent SEM images of TCSE (*f*_w_ = 50%) assemblies.

Given the pronounced differences in TCSE properties between aqueous and organic media, we further examined its fluorescence kinetics in THF/H_2_O mixtures ([TCSE] = 1.0 × 10^−5^ M). As shown in Fig. 3a, samples with water fractions (*f*_w_, v/v) of 38–50% initially displayed blue emission, with only minor decreases in intensity and slight red shifts upon increasing water content. Remarkably, the emission color evolved over time in a water-content-dependent manner: for *f*_w_ = 45% and 50%, the emission converted from blue to green within 8 h (Fig. 3b), whereas samples with *f*_w_ = 38–42% remained blue. After 24 h, however, all samples in the 38–50% range exhibited green emission (Fig. 3c). These results indicate that higher water content accelerates color evolution, likely because stronger hydrophobic environment facilitates faster assembly into nanorods.

To elucidate the underlying mechanism, *f*_w_ = 50% was selected as a representative case. Over 4 h, the absorption spectra broadened, with the ∼340 nm peak decreasing and a shoulder at ∼410 nm emerging (Fig. 3d). Concurrently, pronounced Tyndall scattering developed (Fig. 3d, inset), confirming time-dependent self-assembly. Concurrently, the normalized fluorescence spectra displayed a ∼50 nm red shift (Fig. 3e), consistent with the gradual color transition from blue to green (Fig. 3e, inset). This spectral evolution was further corroborated by the CIE 1931 chromaticity coordinates, which shifted from the blue to the green region within 4 h (Fig. 3f).

To further elucidate the influence of water content on the aggregation behavior of TCSE, samples with *f*_w_ = 45% and 100% were investigated. At *f*_w_ = 45%, the absorption spectra gradually broadened over 8 h, with the ∼340 nm peak decreasing and the 410 nm region increasing, accompanied by the emergence of a pronounced Tyndall effect (Fig. S11a, SI). Correspondingly, the fluorescence spectra display a progressive bathochromic shift of approximately 51 nm, with the emission color evolving from blue to green (Fig. S11b, SI). For systems with *f*_w_ = 45% and 50%, the fluorescence lifetimes increase continuously over time (Fig. S12 and Table S3, SI), while the absolute fluorescence quantum yields show a slight decrease (Fig. S13 and Table S3, SI). In contrast, at *f*_w_ = 100%, the absorption profiles remain essentially unchanged over 12 h, although strong Tyndall scattering persists (Fig. S11c, SI). Both the fluorescence intensity and emission color are maintained, exhibiting stable bright green luminescence (Fig. S11d, SI). Collectively, these results indicate that water content governs not only the aggregation kinetics of TCSE but also its associated fluorescence evolution.

Compared with molecular systems containing one or two CS units, the TCS framework is expected to provide a larger π-conjugated surface, stronger intermolecular π–π interactions, and greater packing complexity (Table S5, SI). These structural characteristics may enable access to multiple supramolecular packing states with distinct molecular organizations and optical properties, thereby making time-evolving fluorescence possible. Morphological studies provided further insight. At 50% water, freshly prepared TCSE formed irregular nanoparticles, regarded as metastable intermediates, which evolved into disordered aggregates (2 h) and eventually into well-ordered nanorods after 4 h (Fig. 3g). In 100% water, by contrast, both fresh and aged samples consistently displayed nanorod morphologies (Fig. S14, SI), confirming nanorods as the thermodynamic product. Notably, freshly prepared nanoparticles at 50% water exhibited blue emission similar to that in pure THF, indicating that the color change originates from π–π stacking, most likely associated with the onset of extended conjugation and charge transfer in the ordered assemblies. This observation is corroborated by the preceding theoretical calculations and emission spectra in different solvents.

The time-dependent fluorescence, UV-vis absorption, and SEM observations consistently reveal a continuous structural evolution from nanoparticles to nanorods rather than direct precipitation. Furthermore, temperature-dependent fluorescence measurements show that elevating the temperature markedly accelerates this fluorescence evolution, indicating that thermal energy facilitates structural reorganization of the initial assemblies (Fig. S15 and S16, SI). After cooling back to room temperature and aging for 24 h, the green emission remained stable without reverting to blue (Fig. S17, SI). These observations are consistent with a transformation from an initially metastable assembly toward a thermodynamically more stable nanorod state.

**Fig. 4:**
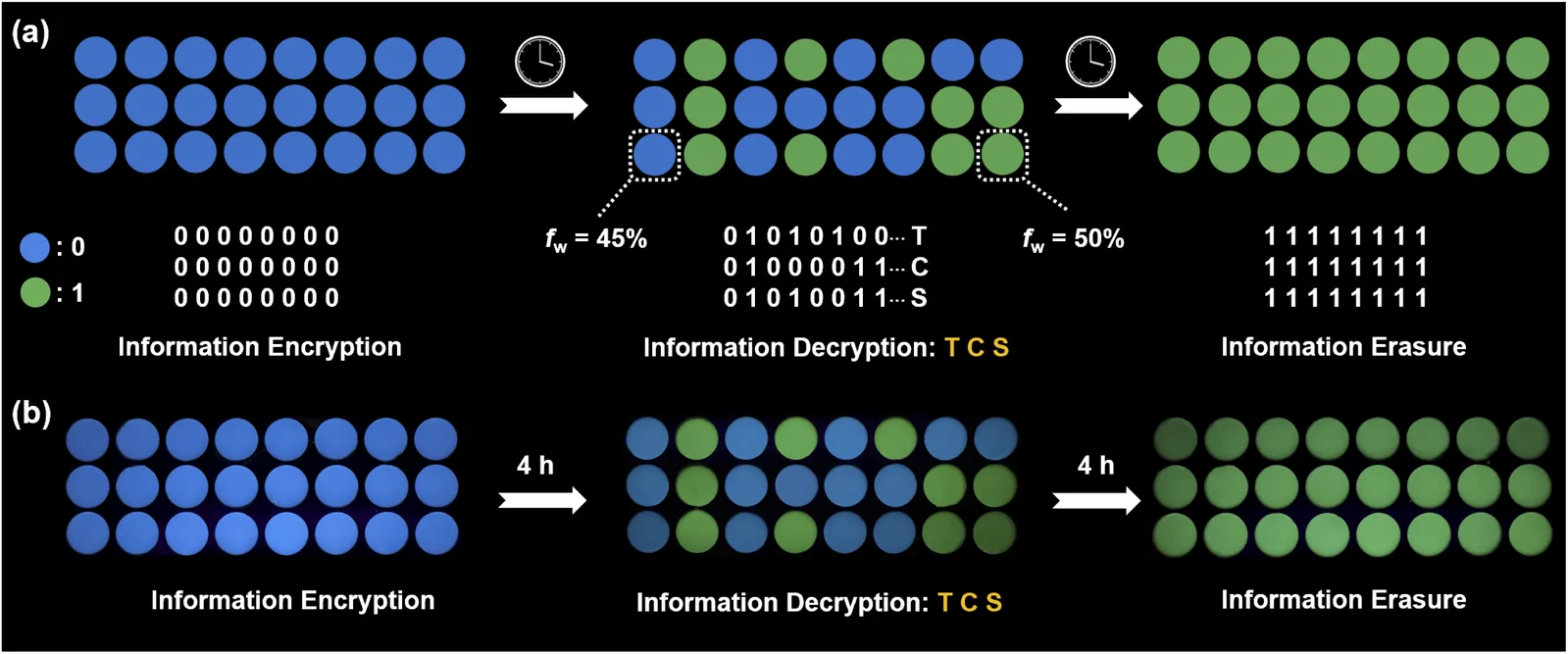
(a) Conceptual illustration of the time-dependent information encryption/decryption strategy based on binary coding. (b) Fluorescence images validating temporal binary coding for data anticounterfeiting, constructed from TCSE samples with water fractions of 50% (rapid transition) and 45% (slow transition). [TCSE] = 1.0 × 10^−5^ M.

The development of advanced optical materials with time-dependent responses is critically important for next-generation high-security data protection and anticounterfeiting. Such materials not only enable multidimensional and multilevel encryption but also significantly enhance resistance to unauthorized decryption or replication during storage and transmission. Given that the fluorescence color transition of TCSE in solution (blue → green) can be precisely tuned by water content, we devised a time-dependent encryption system based on a single-component fluorophore. By integrating this time-resolved fluorescence behavior with binary coding, we established an encryption strategy that exploits both temporal control and color differentiation. Specifically, color-switchable fluorescent dot matrices were employed to encode and decode information within predefined time windows (Fig. 4a). In this system, blue-emitting dots represent binary “0”, while time-evolved green emission corresponds to binary “1”.

As shown in Fig. 4b, TCSE solutions with water fractions of 50% and 45% were selected for encoding due to their favorable fluorescence performance and distinct color-transition kinetics. Notably, both solutions were colorless and transparent under daylight and showed no visible changes over time (Fig. S18, SI). Initially, all dots emitted blue fluorescence, corresponding to a blank encrypted state. After 4 h, a fraction of the dots (*f*_w_ = 50%) gradually converted to green, unveiling the encrypted message “TCS” through fluorescence color differentiation. Upon further aging to 8 h, the remaining blue dots (*f*_w_ = 45%) also transitioned to green, thereby automatically erasing the message. Notably, the lifetime of the metastable emissive state can potentially be regulated by tuning the assembly kinetics. For example, varying the water fraction in the THF/H_2_O mixture may accelerate or retard the nanoparticle-to-nanorod transition by modulating the hydrophobic driving force and molecular rearrangement process, thereby providing a feasible strategy for controlling the temporal window of information encoding and decoding. Owing to the single-component nature of TCSE, the material can be readily recovered without additional purification. Therefore, TCSE enables time-dependent, erasable fluorescence encoding, allowing information to be encrypted and decrypted at a precise time, before being automatically erased within defined time windows.

**Fig. 5:**
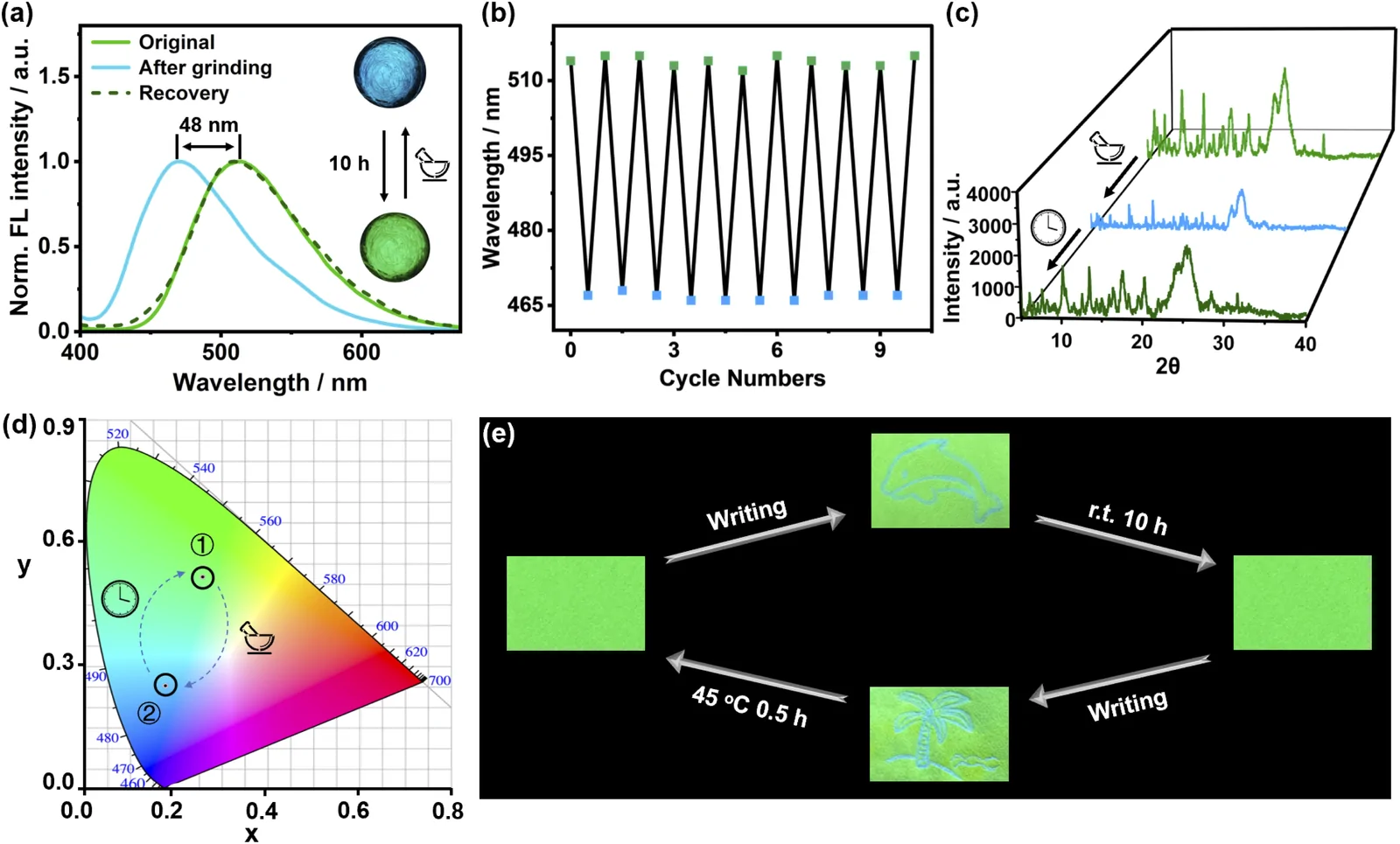
(a) Normalized solid-state fluorescence spectra of TCSE before grinding, after grinding, and after spontaneous recovery (dashed curve). Inset: corresponding fluorescence images of TCSE powder. (b) Plot showing reversible switching in fluorescence emission color during cycling between grinding and recovery. (c) XRD patterns of TCSE powder in the pristine, ground, and self-recovered states. (d) CIE 1931 chromaticity coordinates of TCSE powder before and after grinding. (e) Reversible fluorescence “writing” and “erasing” on a TCSE-impregnated paper.

We further investigated TCSE in the solid state to explore its potential for self-erasable fluorescent writing. Owing to its pronounced AIE characteristics, pristine TCSE powder exhibits intense green fluorescence. Interestingly, mechanical grinding induces a pronounced blue shift in the emission (Fig. 5a), with the fluorescence maximum shifting from 518 to 470 nm, consistent with disruption of the ordered molecular packing. Strikingly, the blue-emissive powder spontaneously recovers its original green emission within 10 h, suggesting a self-healing process of the solid-state packing. This reversible mechanochromic behavior can be repeated for at least ten grinding–recovery cycles without noticeable fatigue (Fig. 5b).

X-ray diffraction (XRD) analysis further reveals the structural origin of this fluorescence switching. As shown in Fig. 5c, the pristine powder exhibits sharp and intense diffraction peaks, characteristic of a highly crystalline structure. Upon grinding, the diffraction peaks are markedly weakened, indicating a transition from a crystalline to an amorphous state. After spontaneous recovery, the sharp diffraction peaks reappear, demonstrating the restoration of crystallinity. Consistently, SEM images reveal that the well-defined nanorod morphology of the pristine powder collapses into irregular nanoparticles after grinding and is subsequently reconstructed after spontaneous recovery (Fig. S19, SI), providing direct morphological evidence for the reversible structural transformation.

To further support this structural interpretation, we measured the fluorescence lifetimes and absolute quantum yields of TCSE powder before grinding, after grinding, and after spontaneous recovery. Upon grinding, the fluorescence lifetime decreases from 13.30 to 10.89 ns, whereas the absolute quantum yield increases from 23.04% to 28.53%, excluding enhanced non-radiative decay as the origin of the blue shift. After spontaneous recovery, both the fluorescence lifetime (13.01 ns) and quantum yield (23.45%) return to values nearly identical to those of the pristine sample (Table S4, Fig. S20 and S21, SI). Together with the reversible changes in crystallinity and morphology, these results indicate that the grinding-induced blue shift mainly originates from reversible changes in molecular packing associated with crystallinity, rather than significant changes in molecular conformation.

Notably, this reversible fluorescence evolution closely parallels the time-dependent emission changes observed during solution self-assembly. In aqueous solution, TCSE evolves from blue-emissive monomeric or weakly associated species into green-emissive nanorod assemblies. Likewise, in the solid state, mechanical grinding converts the green-emissive crystalline powder into a blue-emissive amorphous state, which spontaneously recovers to the original crystalline phase over time. The close correspondence between these two fluorescence evolution pathways suggests that the ground amorphous state possesses a packing environment resembling that of the monomeric or initially assembled species in solution, whereas the recovered crystalline state shares similar intermolecular packing characteristics with the emissive nanorod assemblies formed in water. Taken together, these results establish a clear correlation between the solution-state assembly pathway and the solid-state structural transformation, providing a unified structural origin for the reversible fluorescence evolution of TCSE, as further supported by the CIE 1931 chromaticity analysis (Fig. 5d).

Exploiting this robust and self-recovering mechanochromism, we fabricated rewritable fluorescent paper by coating TCSE onto filter paper. The pristine paper displays bright green fluorescence, and patterns can be written by applying localized pressure (*e.g.*, a “dolphin”), generating blue-emitting information against the green background (Fig. 5e). Remarkably, the written patterns spontaneously disappear within 10 h without external intervention, owing to the gradual molecular rearrangement from the mechanically disrupted state back to the thermodynamically more favored ordered packing structure. Control experiments under a dry N_2_ atmosphere showed that the ground powder recovered to the original green-emissive state with a recovery time nearly identical to that observed in air (Fig. S22), indicating that neither oxygen nor moisture is essential for the recovery process. This result further supports that the spontaneous fluorescence recovery is governed primarily by reversible molecular packing rearrangement from the metastable amorphous state to the thermodynamically stable crystalline state.

The erasure process can be further accelerated by mild heating (45 °C, 30 min) or exposure to solvent vapor (DCM, 3 min), which enhance molecular mobility and facilitate the reorganization of TCSE assemblies (Fig. 5e, S23 and S24, SI). Specifically, thermal activation promotes molecular rearrangement within the solid matrix, whereas DCM vapor transiently increases molecular mobility through solvent-induced swelling, enabling rapid recovery of the original emissive state. Furthermore, in addition to DCM, exposure of the ground powder to other solvent vapors, including chloroform, tetrahydrofuran, and acetonitrile, also rapidly restored the original green emission within minutes (Fig. S25, SI). These results indicate that the accelerated recovery is not specific to DCM but is a general consequence of enhanced molecular mobility induced by solvent vapors, which facilitates the structural rearrangement required for fluorescence recovery. Conversely, the lifetime of the written information can potentially be prolonged by suppressing molecular mobility, for example through low-temperature storage or molecular engineering strategies that increase the kinetic barrier for structural rearrangement. Indeed, storing the ground powder at −15 °C significantly prolonged the recovery time from approximately 10 h at room temperature to about 30 h (Fig. S22, SI), demonstrating that restricted molecular mobility effectively retards the structural reorganization. This result provides direct experimental evidence that the information lifetime can be deliberately extended, complementing the accelerated erasure achieved by heating or solvent-vapor treatment. Collectively, these results demonstrate the feasibility of bidirectional temporal control over the lifetime of stored information, highlighting the potential of TCSE for dynamic information storage, anti-counterfeiting, and information processing.

## Conclusions

In summary, we have developed a single-component supramolecular system that integrates time-evolving self-assembly with dynamic optical outputs for temporal information processing. The tris(cyanostyryl)benzene derivative TCSE undergoes a spontaneous assembly pathway in THF/H_2_O mixtures, evolving from metastable blue-emissive nanoparticles to thermodynamically more favored green-emissive nanorods. This time-resolved structural transformation intrinsically generates dual-color fluorescence and enables temporal information encryption without requiring multiple components or external stimuli. In the solid state, TCSE further exhibits reversible mechanofluorescence arising from crystalline–amorphous transitions, allowing the fabrication of rewritable fluorescent media capable of autonomous information erasure. By coupling assembly kinetics with tunable emission states within a single molecular platform, this work establishes a general strategy for constructing temporally programmable luminescent materials. Such time-evolving supramolecular systems may open new opportunities for dynamic photonic devices, high-security information storage, and advanced anticounterfeiting technologies.

## Author contributions

Q. Z. synthesized and characterized the molecules, constructed the self-assembled systems, and performed the photophysical measurements. Q. L. measured the SEM. L. W. revised the manuscript. T. X. designed the work, provided intellectual input, and wrote and revised the manuscript.

## Conflicts of interest

There are no conflicts to declare.

## Supplementary Material

SC-OLF-D6SC04670C-s001

## Data Availability

All the data supporting this article have been included in the main text and the supplementary information (SI). Supplementary information: synthetic details, additional NMR spectra, and HR-ESI-MS spectra of individual compounds. See DOI: https://doi.org/10.1039/d6sc04670c.
