# Supplementary material for: Time-evolving self-assembly of a single-component fluorophore enables dynamic emission for temporal information encryption and self-erasing writing
Source: Chem Sci. 2026 Aug 25;17(40):19533–40. doi: 10.1039/d6sc04670c (PMC13532919; doi:10.1039/d6sc04670c)
Supplement: Supplementary file 1 [file SC-017-D6SC04670C-s001.pdf]

Supplementary Information (SI) for Chemical Science.
This journal is © The Royal Society of Chemistry 2026

Supporting Information

Time-evolving self-assembly of a single-component fluorophore enables dynamic emission for temporal information encryption and self-erasing writing

Qiaona Zhang[1], Qing Liu[1], Leyong Wang[2], and Tangxin Xiao[1,*]

[1] School of Petrochemical Engineering, Changzhou University, Changzhou 213164, China

[2] School of Chemistry, Nanjing University, Nanjing 210023, China

*Corresponding Author: xiaotangxin@cczu.edu.cn

Table of contents

1. Materials, instruments and abbreviations S2
2. Synthesis of **TCSE** S3
3. Solvent effects and theoretical calculations of **TCSE** S7
4. Time-dependent self-assembly and photophysical properties S11
5. Information encryption based on binary code S15
6. Self-erasable writing materials S15
7. Comparison of **TCSE** with other CS systems S19
8. References...S20

1. Materials, instruments and abbreviations

All reagents were purchased from commercial suppliers and used as received unless otherwise indicated. The nuclear magnetic resonance (NMR) spectra were acquired using a Bruker AVANCE III (400 MHz/600 MHz) spectrometer (Bruker Corporation, Billerica, Massachusetts, United States). High-resolution electrospray ionization mass spectra (HR-ESI-MS) were recorded on an Agilent Technologies 6540 UHD Accurate-Mass system (Agilent Technologies, Inc., Santa Clara, California, United States). UV-vis absorption spectra were measured on a Shimadzu UV-1900i UV-visible spectrometer (Shimadzu Corporation, Kyoto, Japan). Fluorescence measurements were performed on an Agilent Cary Eclipse spectrofluorometer (G9800A) (Agilent Technologies, Inc., Santa Clara, California, United States). Fluorescence lifetimes were measured employing time-correlated single photon counting on an FLS980 instrument (Edinburgh Instruments, Livingston, United Kingdom) equipped with a pulsed xenon lamp. Analysis of fluorescence decay curves was subjected to fit a mono- or bi-exponential decay. The instrument response function (IRF) measures the scattering of laser excitation from non-fluorescent control samples to determine the fastest possible response of the detectors. The quantum yields were measured on an FLS980 instrument (Edinburgh Instruments, Livingston, United Kingdom) equipped with an integrating sphere. Scanning electron microscope (SEM) investigations were carried out on a SUPRA-55 instrument (Carl Zeiss AG, Oberkochen, Germany). Transmission electron microscope (TEM) investigations were carried out on a JEM-2100 instrument (JEOL Ltd., Akishima, Tokyo, Japan). X-ray diffractometer (XRD) patterns were measured on a Rigaku Corporation D/MAX 2500 diffractometer (Rigaku Corporation, Akishima, Tokyo, Japan). The CIE (Commission Internationale de l'Eclairage) 1931 chromaticity coordinates were calculated from the emission spectra. Abbreviations: DCM = dichloromethane; DMF = *N*,*N*-dimethylformamide; EA = ethyl acetate; THF = tetrahydrofuran; M = mol/L; PCC = pyridinium chlorochromate.

2. Synthesis of TCSE

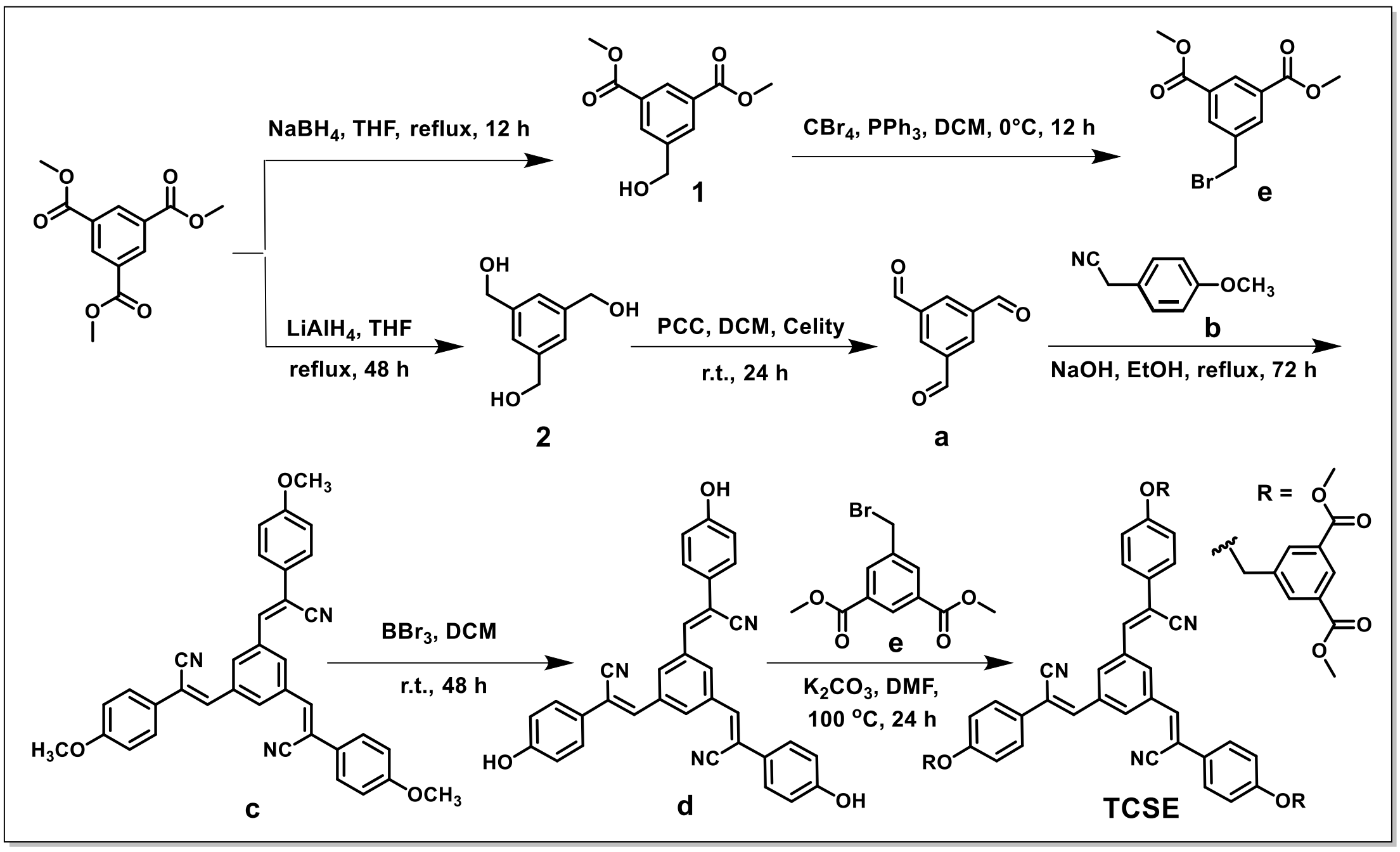

Scheme S1. Synthetic route of **TCSE**.

The synthetic pathway toward **TCSE** is outlined in Scheme S1. Trimethyl 1,3,5-benzenetricarboxylate was first reduced with $NaBH_4$ in refluxing THF to afford dihydroxy intermediate **1**. Bromination of **1** with CBr_4/PPh_3 in DCM at 0 °C produced intermediate **e**. In a parallel sequence, reduction of the same starting material with $LiAlH_4$ furnished tri-hydroxy compound **2**, which was subsequently oxidized by PCC in DCM to yield aldehyde **a**. A base-catalyzed Knoevenagel condensation between **a** and 4-methoxybenzyl cyanide (**b**) afforded π-extended intermediate **c**, followed by demethylation with BBr_3 to give phenolic derivative **d**. Finally, nucleophilic substitution between **d** and **e** in the presence of K_2CO_3 in DMF at 100 °C delivered the target compound, **TCSE**. Compounds **1**, **2**, **a**, and **c-e** were prepared following the literature procedures.[S1-3]

Synthesis of compound c: A mixture of triformylbenzene (1.00 g, 6.2 mmol), *p*-methoxyphenylacetonitrile (3.02 g, 20.5 mmol), and NaOH (1.51 g, 37.8 mmol) in ethanol (150 mL) was stirred at room temperature for 72 h. After completion, the reaction mixture was filtered under reduced pressure, and the residue was washed with ethanol and dried. The dried solid was dissolved in DCM (20 mL) and poured into ethanol (200 mL) to precipitate the product. Filtration under reduced pressure followed by drying afforded compound **c** as a pale yellow layered solid (1.32 g,

2.4 mmol, 39%). ^{1}H NMR (300 MHz, $CDCl_3$, 298 K): δ (ppm) = 8.32 (s, 3H, =C*H*-), 7.66 (d, J = 8.9 Hz, 6H, Ar-*H*), 7.51 (s, 3H, Ar-*H*), 6.99 (d, J = 8.9 Hz, 6H, Ar-*H*), 3.87 (s, 9H, -CH_3).

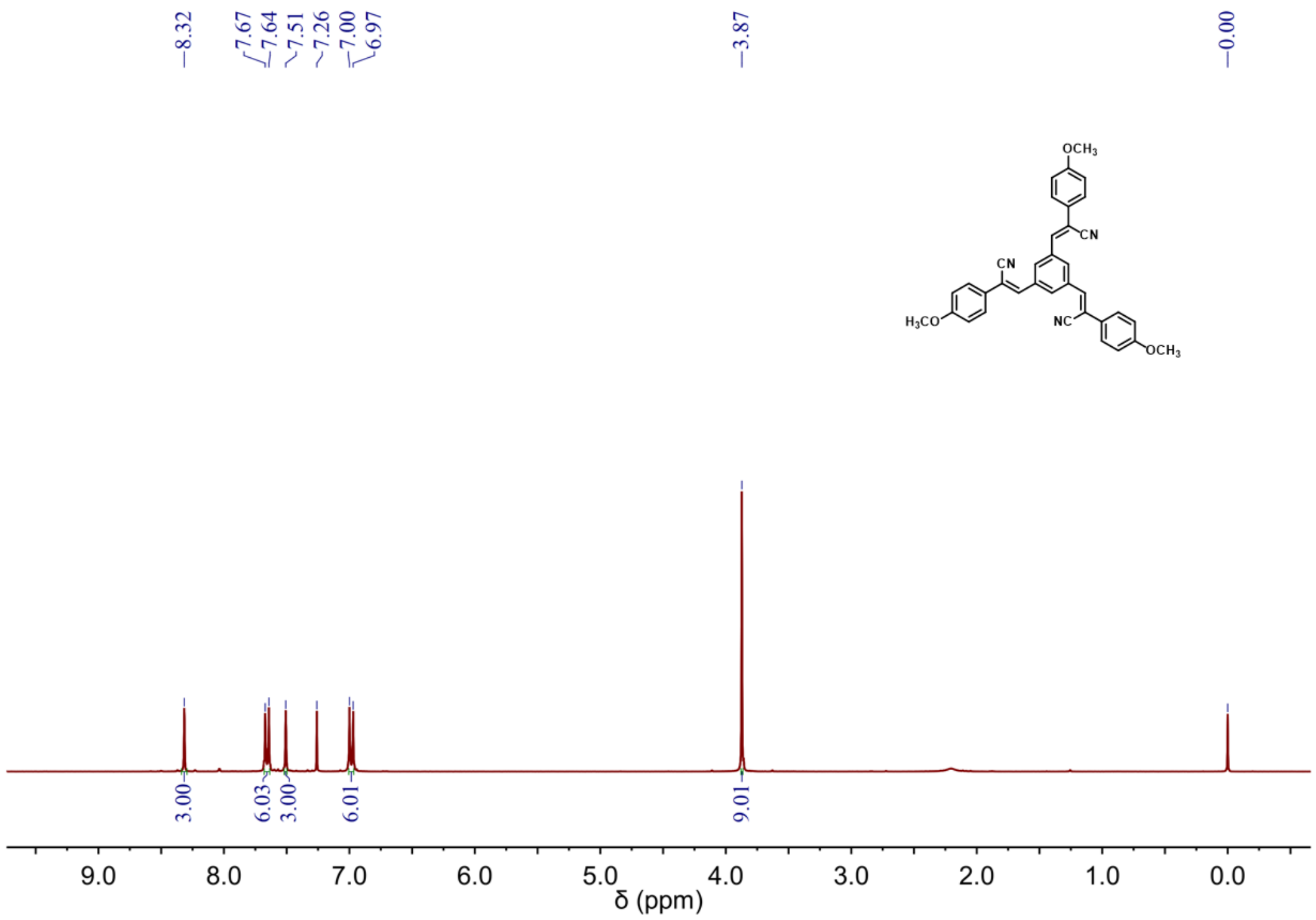

Fig. S1 ^{1}H NMR spectrum (300 MHz, $CDCl_3$, 298 K) of compound **c**.

Synthesis of compound d: A mixture of compound **c** (1.32 g, 2.4 mmol) and dry DCM (40 mL) was placed in a three-neck flask and cooled to 0 °C. BBr_3 (3.16 g, 12.5 mmol) was then added, and the reaction was stirred at room temperature for 48 h. After completion, the reaction was quenched with cold water, and the mixture was extracted with ethyl acetate (3 × 40 mL). The combined organic layers were dried over anhydrous Na_2SO_4, and the solvent was removed under reduced pressure. The crude product was purified by column chromatography (*n*-Hexane/EA = 1:5). The resulting solid was concentrated and dried to afford compound **d** as an orange powder (1.12 g, 2.2 mmol, 92%). ^{1}H NMR (300 MHz, DMSO-d_6, 298 K): δ (ppm) = 10.04 (s, 3H, -O*H*), 8.30 (s, 3H, =C*H*-), 7.96 (s, 3H, Ar-*H*), 7.65 (d, J = 8.7 Hz, 6H, Ar-*H*), 6.92 (d, J = 8.7 Hz, 6H, Ar-*H*).

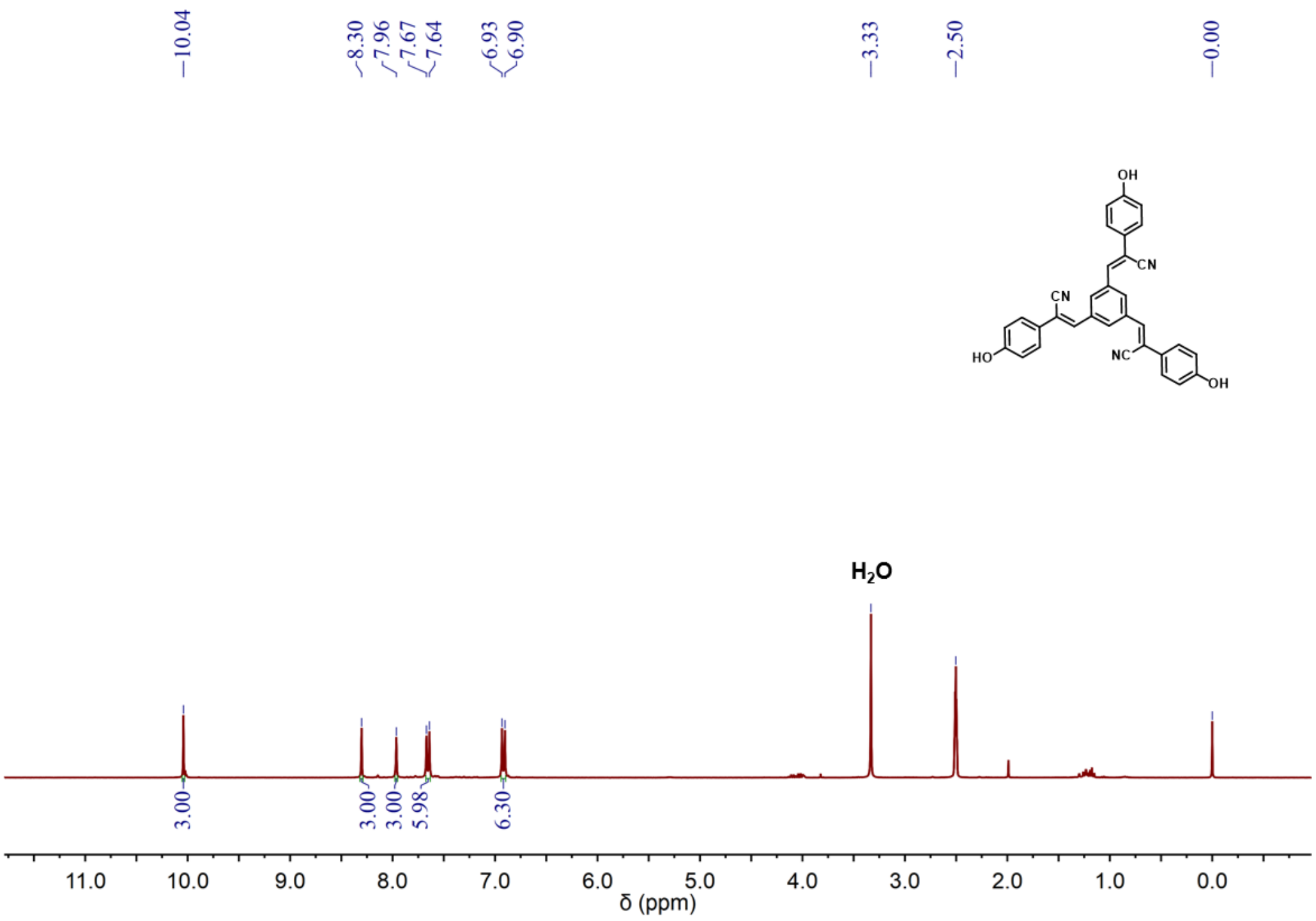

Fig. S2 ^{1}H NMR spectrum (300 MHz, DMSO-d_6, 298 K) of compound **d**.

Synthesis of compound TCSE: A mixture of compound **d** (0.51 g, 1.0 mmol), compound **e** (1.00 g, 3.5 mmol), and K_2CO_3 (2.07 g, 15.0 mmol) in dry acetone (30 mL) was stirred at room temperature for 72 h under nitrogen. After completion, the reaction was diluted with DCM (85 mL), washed with water (3 × 50 mL), and the organic phase was dried over anhydrous Na_2SO_4. The solvent was removed under reduced pressure, and the residue was poured into ethanol (200 mL) to precipitate the product. Filtration afforded **TCSE** as a yellow powder (0.43 g, 0.38 mmol, 38%). ^{1}H NMR (400 MHz, $CDCl_3$, 298 K): δ (ppm) = 8.67 (s, 3H, =C*H*-), 8.33 (s, 9H, Ar-*H*), 7.68 (d, J = 7.9 Hz, 6H, Ar-*H*), 7.53 (s, 3H, Ar-*H*), 7.07 (d, J = 8.3 Hz, 6H, Ar-*H*), 5.21 (s, 6H, -CH_2-), 3.97 (s, 18H, -CH_3). ^{13}C NMR (150 MHz, $CDCl_3$, 298 K): δ (ppm) = 166.0, 159.5, 138.4, 137.6, 135.4, 132.6, 131.2, 130.5, 130.3, 127.8, 127.0, 117.6, 115.5, 113.5, 69.1, 52.5. HR-MS (ESI) *m/z*: $[M + Na]^+$ calcd for $[C_{66}H_{51}N_3NaO_{15}]^+$ = 1148.3213, found 1148.3274.

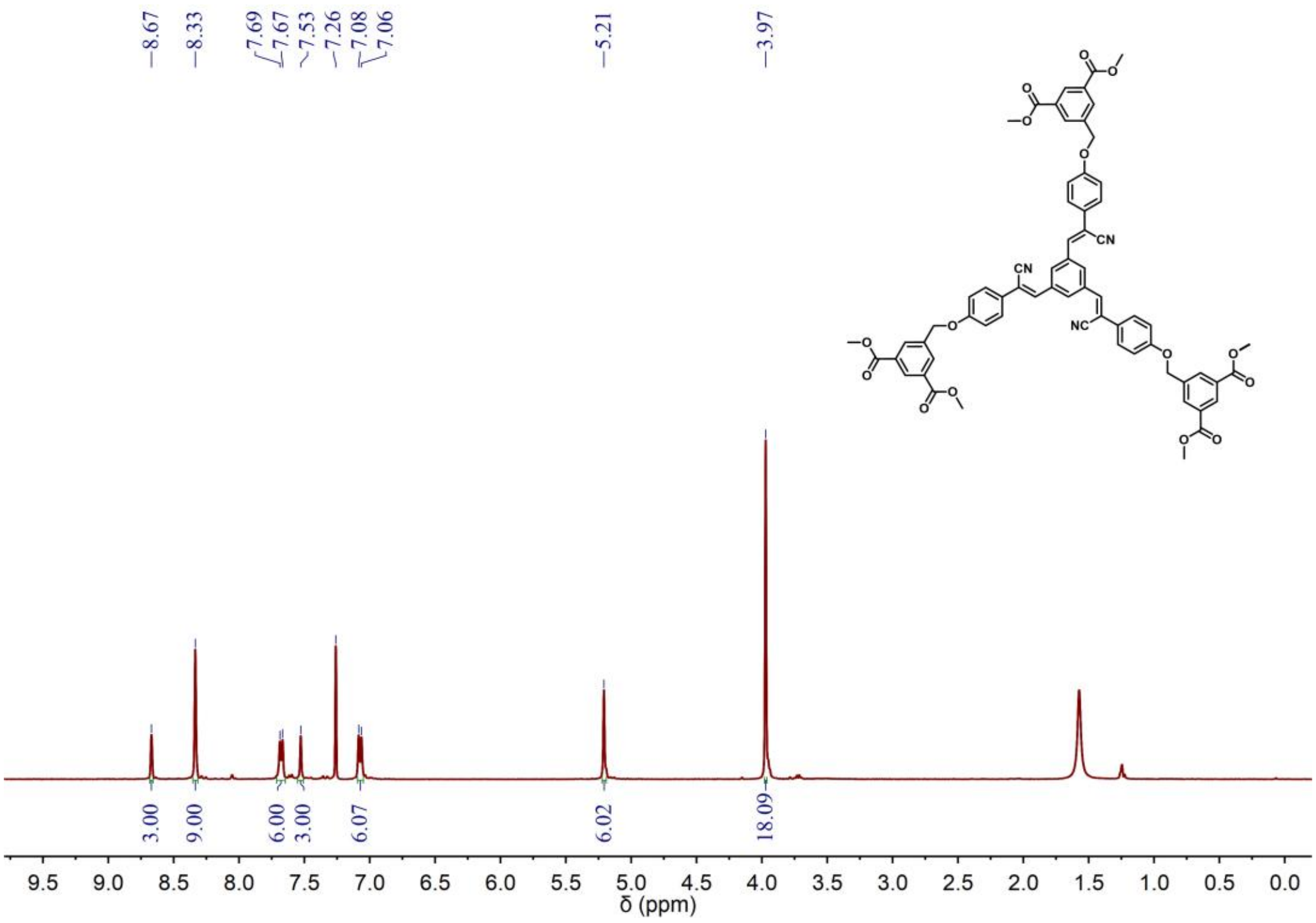

Fig. S3 ^{1}H NMR spectrum (400 MHz, $CDCl_3$, 298 K) of **TCSE**.

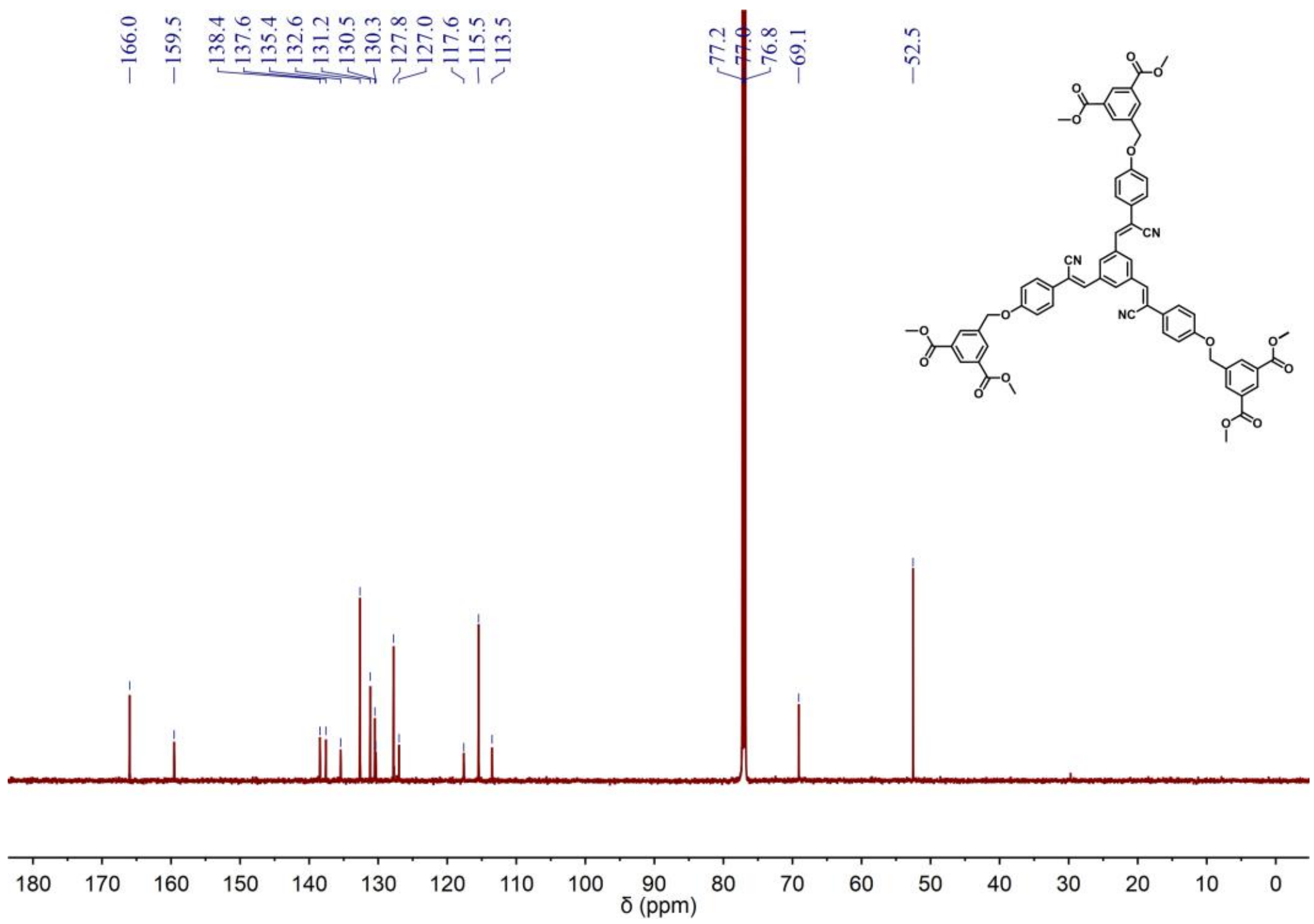

Fig. S4 ^{13}C NMR spectrum (150 MHz, $CDCl_3$, 298 K) of **TCSE**.

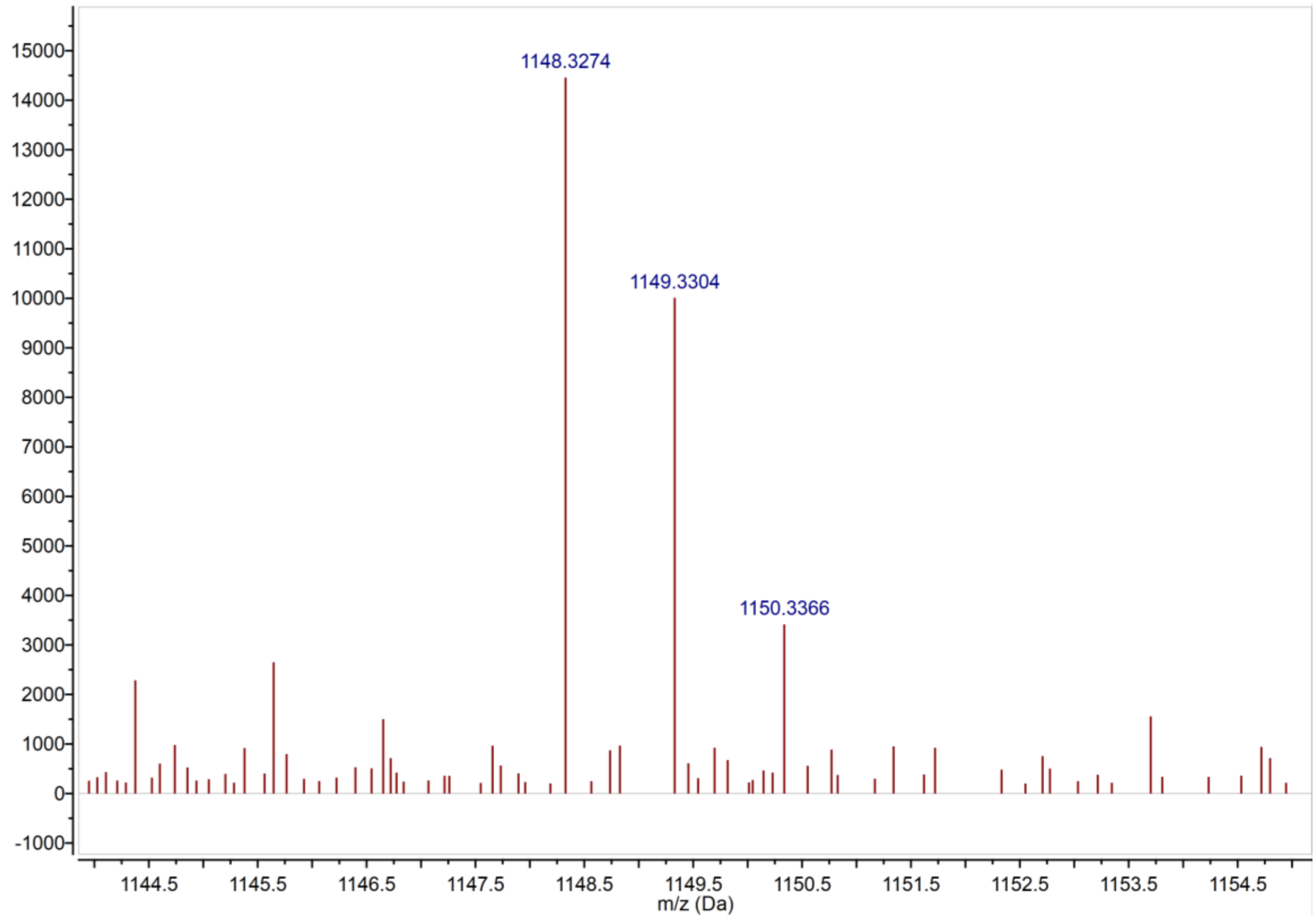

Fig. S5 HR-ESI-MS spectrum of **TCSE**.

3. Solvent effects and theoretical calculations of TCSE

Table S1. Photophysical properties of **TCSE** in different solvents, [**TCSE**] = 1.0 × 10^{-5} M.

Sample	λ_{em}/nm	τ/ns	$\Phi_{f(abs)}$	k_r/ns^{-1}	k_{nr}/ns^{-1}
DCM	428	4.4889	33.88%	0.0755	0.1472
THF	440	5.8116	29.29%	0.0544	0.1217
MeCN	455	4.4687	26.66%	0.0597	0.1641
DMSO	468	3.2090	19.24%	0.0600	0.2516
H_2O	512	10.2997	18.83%	0.0183	0.0788

Note: Calculation of the rate constants corresponding to radiative (k_r) and non-radiative (k_{nr}) pathways are as follows: $k_r = \Phi/\tau$ and $k_{nr} = (1-\Phi)/\tau$.

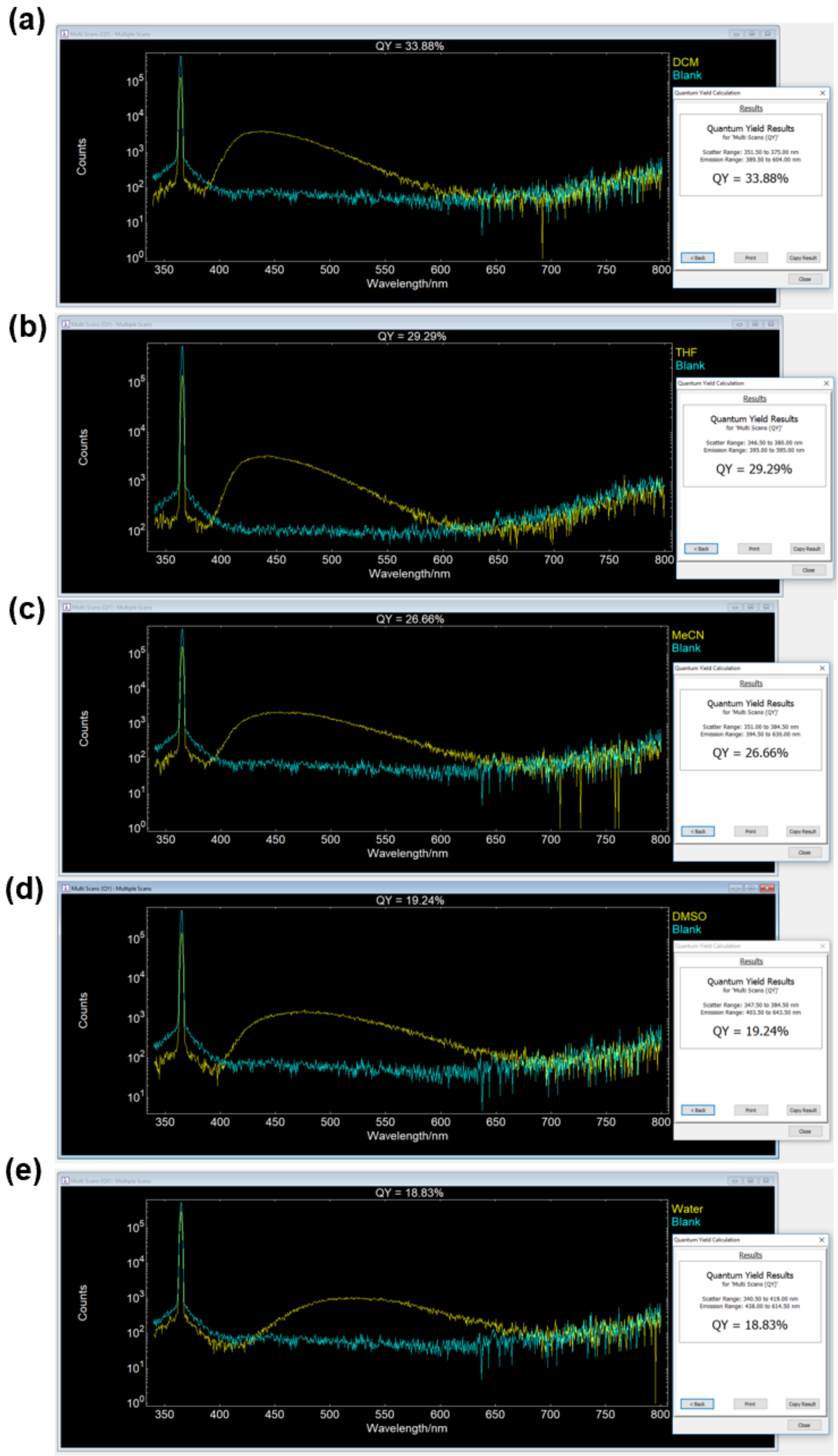

Fig. S6 Absolute fluorescence quantum yields ($\Phi_{f(abs)}$) of **TCSE** in various solvents: (a) DCM, (b) THF, (c) MeCN, (d) DMSO, and (e) H_2O upon excitation at 365 nm, [**TCSE**] = 1.0×10^{-5} M.

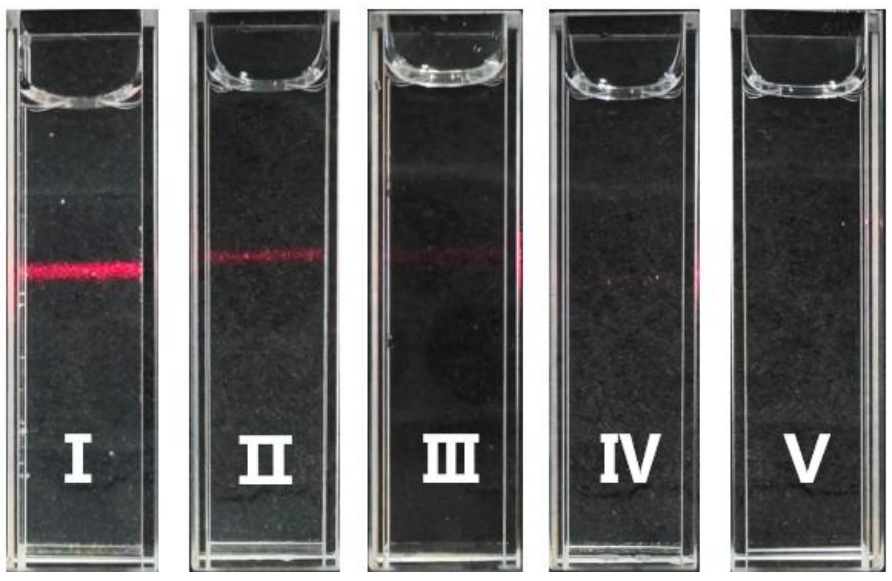

Fig. S7 Tyndall effect of **TCSE** in various solvents: H_2O (I), DMSO (II), MeCN (III), THF (IV), and $CHCl_3$ (V). [**TCSE**] = 1.0×10^{-5} M.

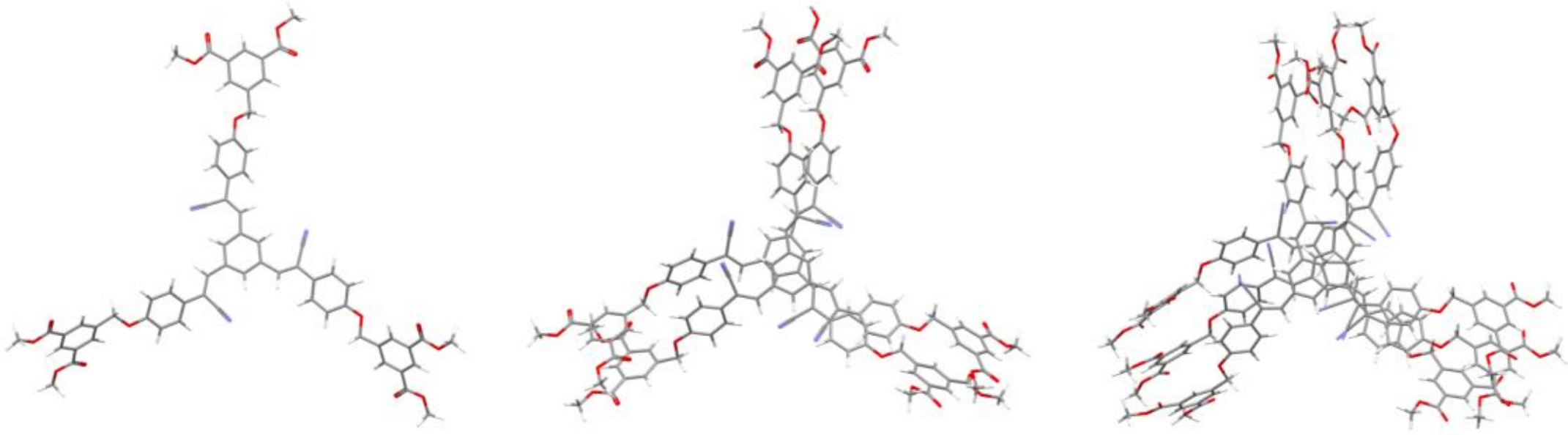

Fig. S8 Optimized structures of the monomer, dimer, and trimer obtained from DFT calculations.

Table S2. Energy gap differences between S_0 and S_1 states, calculated using TDDFT.

Compound	Single Molecule	Dimer	Trimer
ΔE_{0-1}	3.688 eV	3.367 eV	3.163 eV

The geometry optimizations were performed using the B3LYP-D3 functional with the 6-31G* basis set. For excited-state calculations, the CAM-B3LYP-D3 functional paired with the 6-311G** basis set was employed.

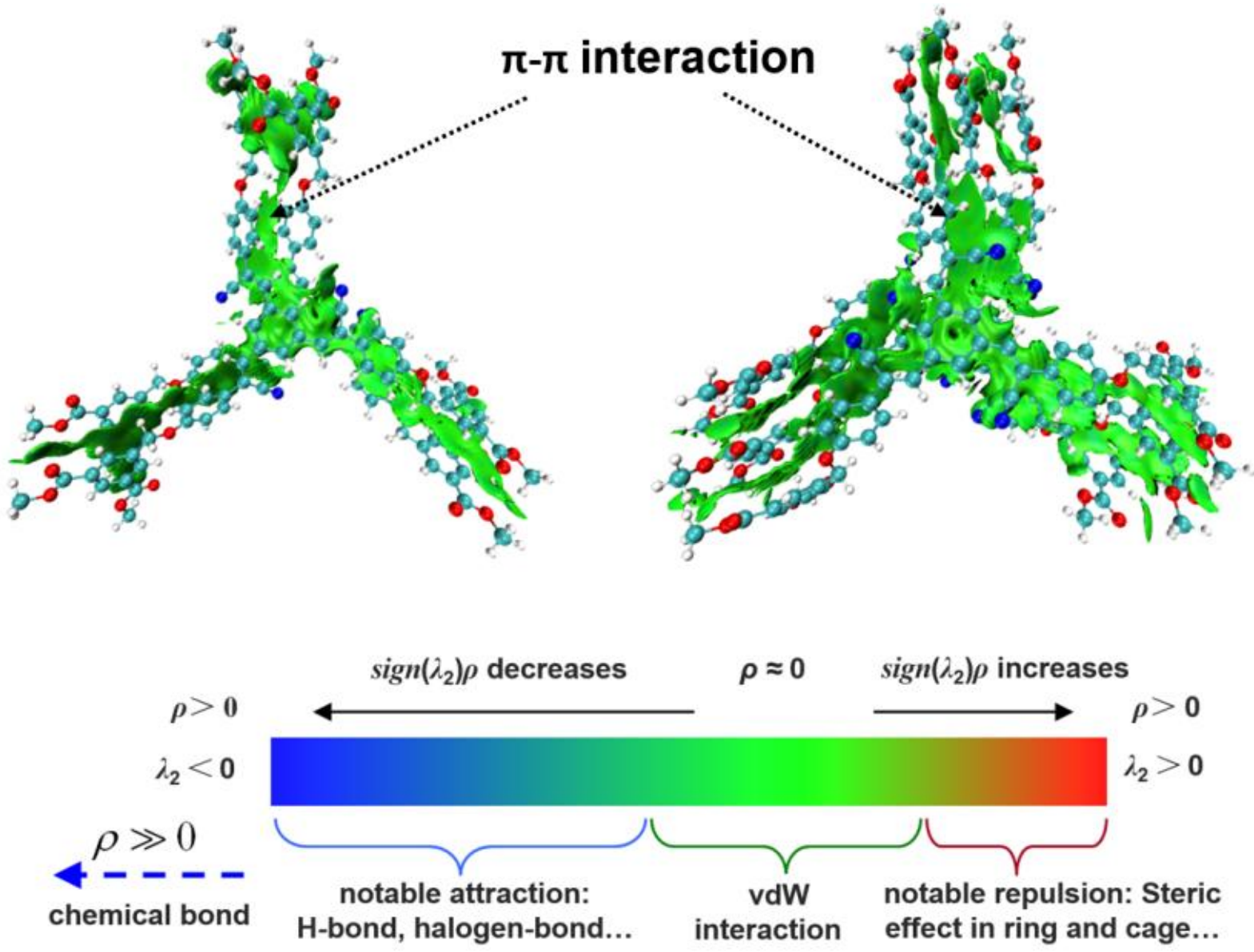

Fig. S9 IGMH analysis of the **TCSE** dimer and trimer highlighting π–π stacking–dominated intermolecular interactions.

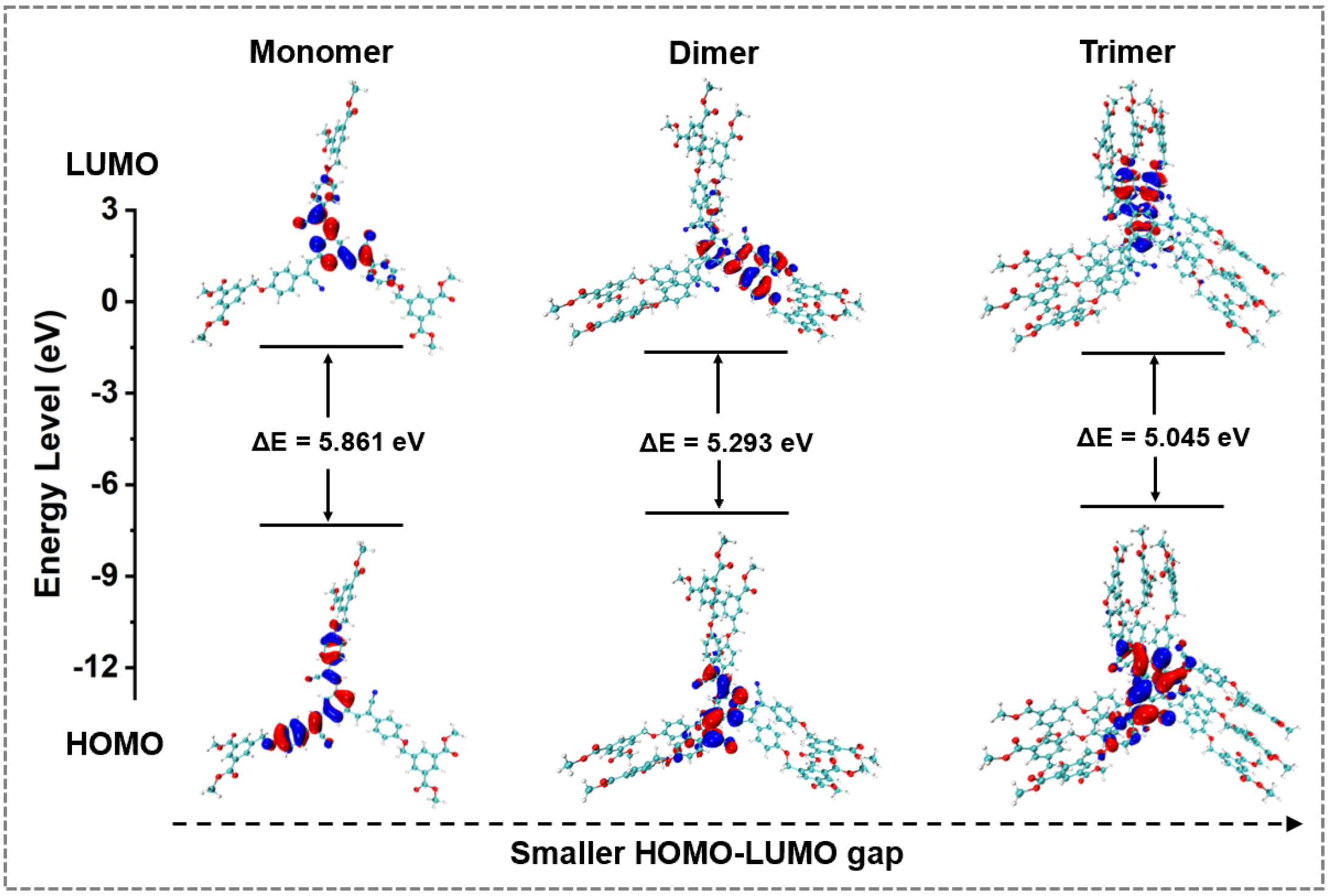

Fig. S10 Frontier molecular orbitals and energy levels of **TCSE** monomer, dimer, and trimer showing aggregation-induced narrowing of the HOMO–LUMO gap.

4. Time-dependent self-assembly and photophysical properties

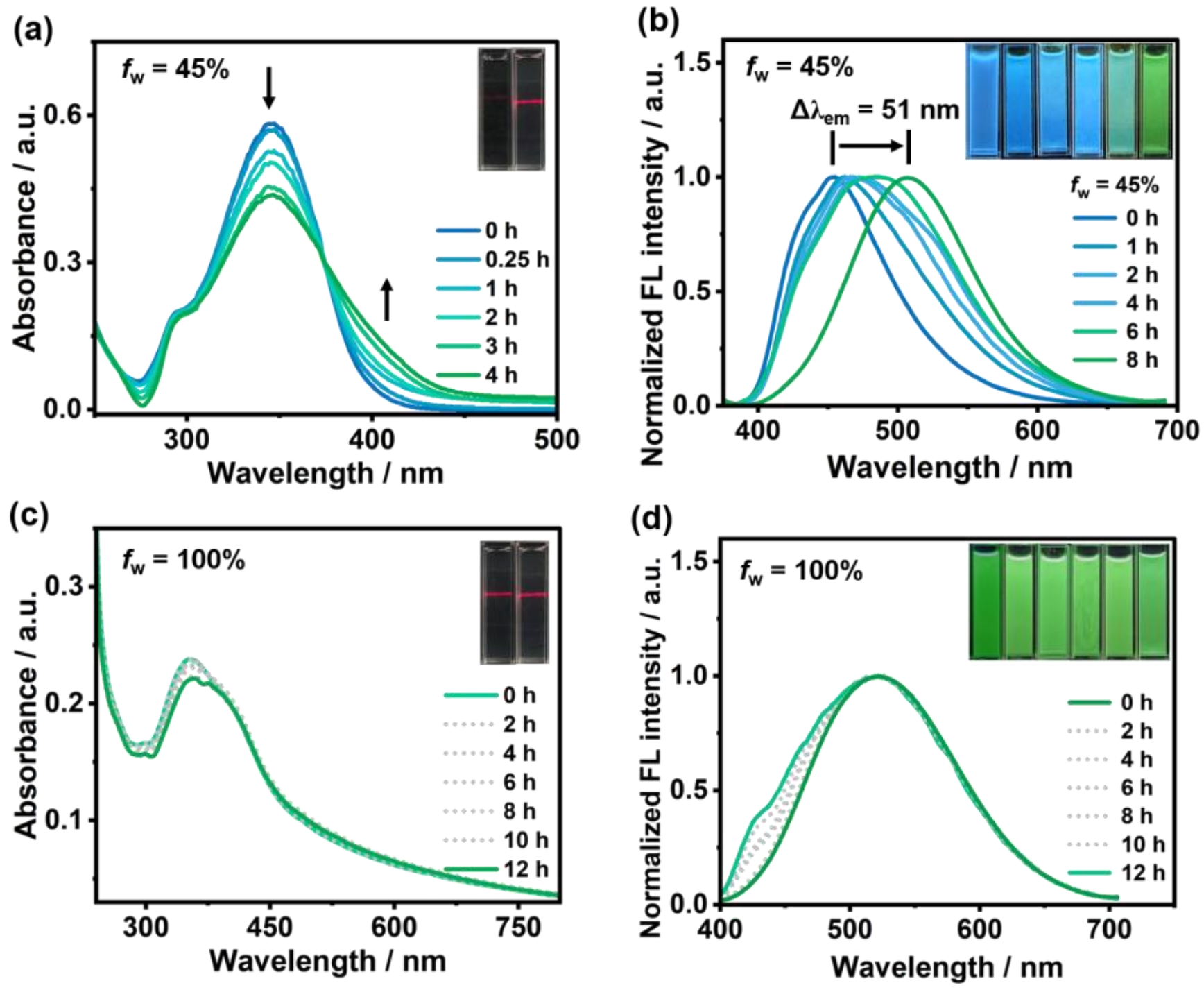

Fig. S11 (a) UV-vis spectra of **TCSE** (f_w = 45%) acquired at different times. Inset: Tyndall effect at 0 h (left) and 8 h (right). (b) Normalized fluorescence spectra of **TCSE** (f_w = 45%) acquired at different times. Inset: fluorescence images of **TCSE** (45%) from 0 to 8 hours. (c) UV-vis spectra of **TCSE** (f_w = 100%) acquired at different times. Inset: Tyndall effect at 0 h (left) and 12 h (right). (d) Normalized fluorescence spectra of **TCSE** (f_w = 100%) acquired at different times. Inset: fluorescence images of **TCSE** (100%) from 0 to 12 h, [**TCSE**] = 1.0 × 10^{-5} M, λ_{ex} = 365 nm.

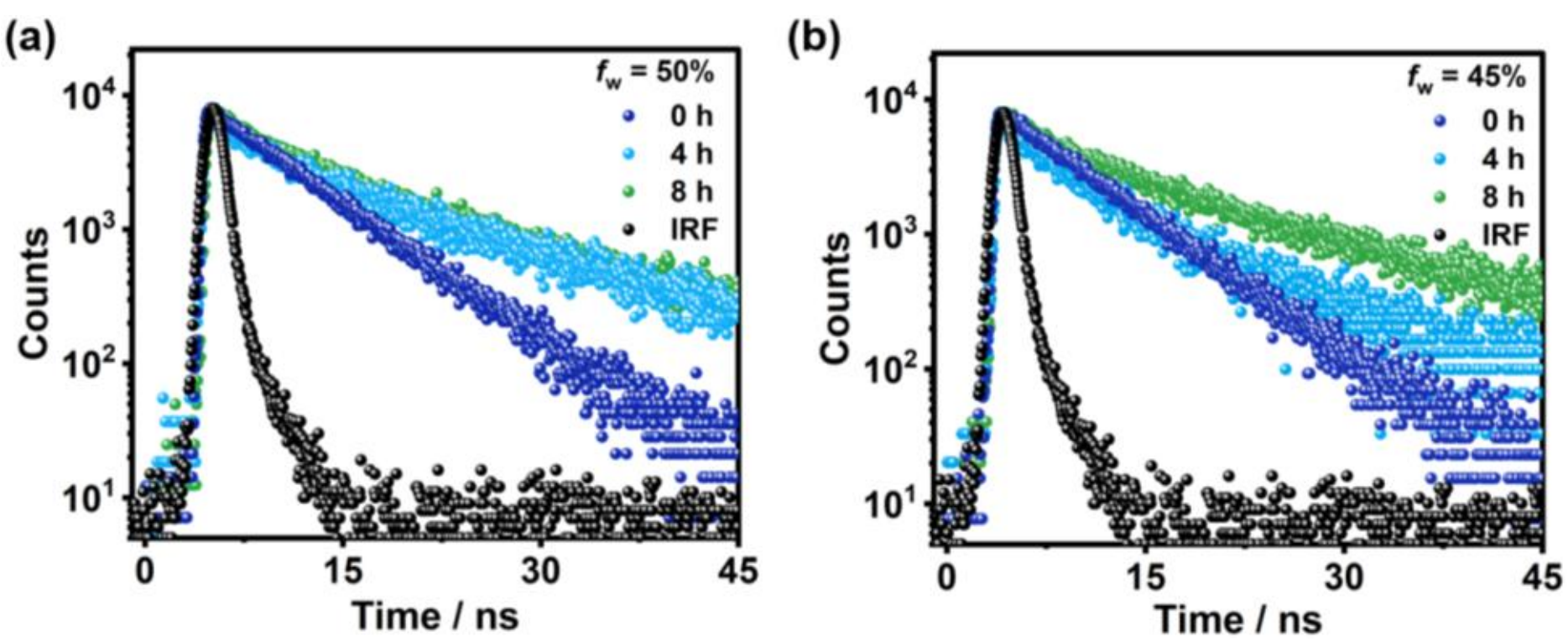

Fig. S12 Fluorescence decay profiles of **TCSE** with different water content at different time. (a) f_w = 50% (b) f_w = 45%. [**TCSE**] = 1 × 10^{-5} M.

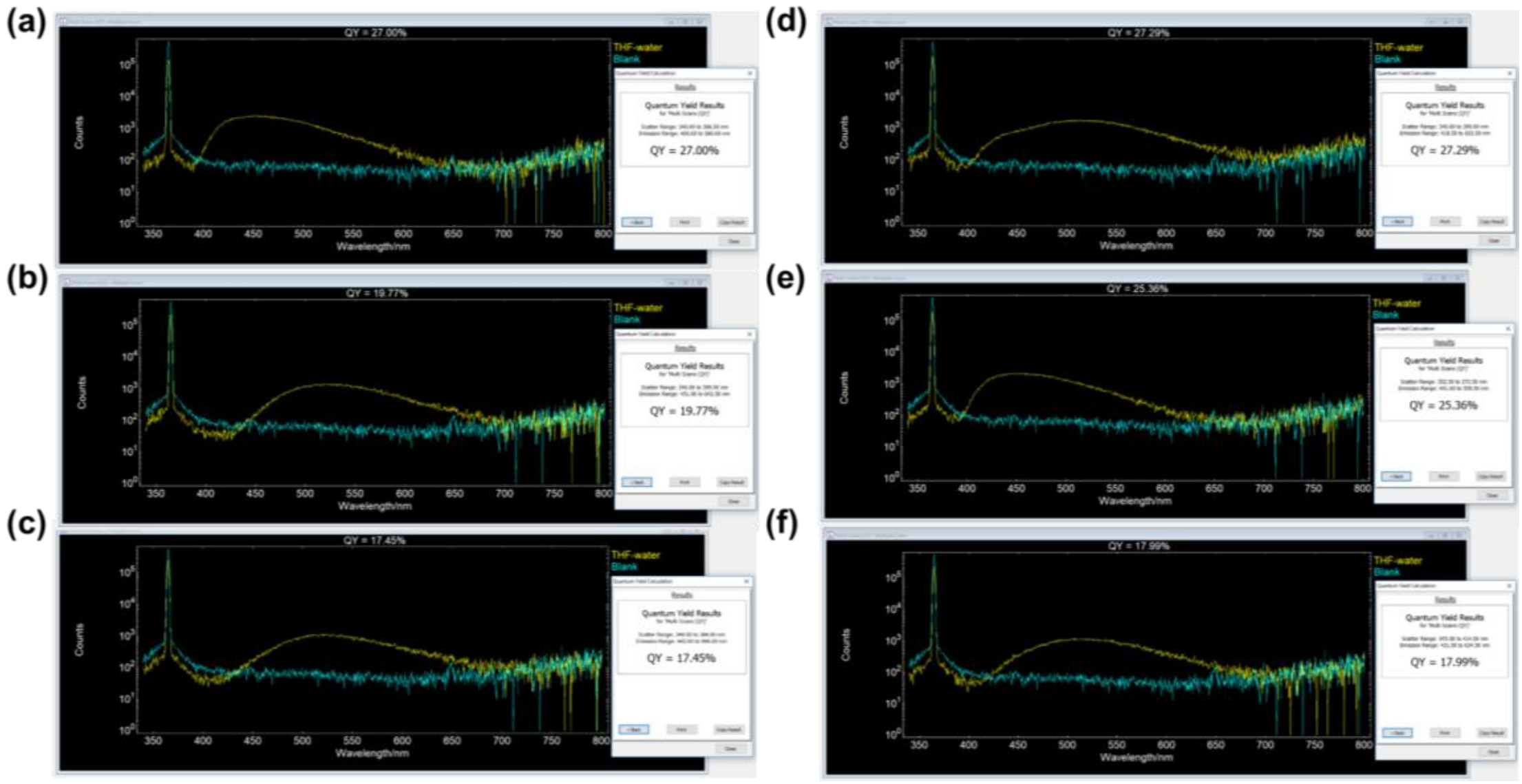

Fig. S13 Absolute fluorescence quantum yields ($\Phi_{f(abs)}$) of **TCSE** with different water content at different times. (a) 0 h (f_w = 50%), (b) 4 h (f_w = 50%), (c) 8 h (f_w = 50%), (d) 0 h (f_w = 45%), (e) 4 h (f_w = 45%), (f) 8 h (f_w = 45%).

Table S3. Fluorescence lifetimes and quantum yields of **TCSE** with different water content at different times.

**Sample**	**Time interval**	**τ/ns**	**$\Phi_{f(abs)}$**
	0 h	6.2807	27.00%
f_w = 50%	after 4 h	8.9434	19.77%
	after 8 h	9.8074	17.45%
	0 h	6.1801	27.29%
f_w = 45%	after 4 h	7.1029	25.36%
	after 8 h	9.8547	17.99%

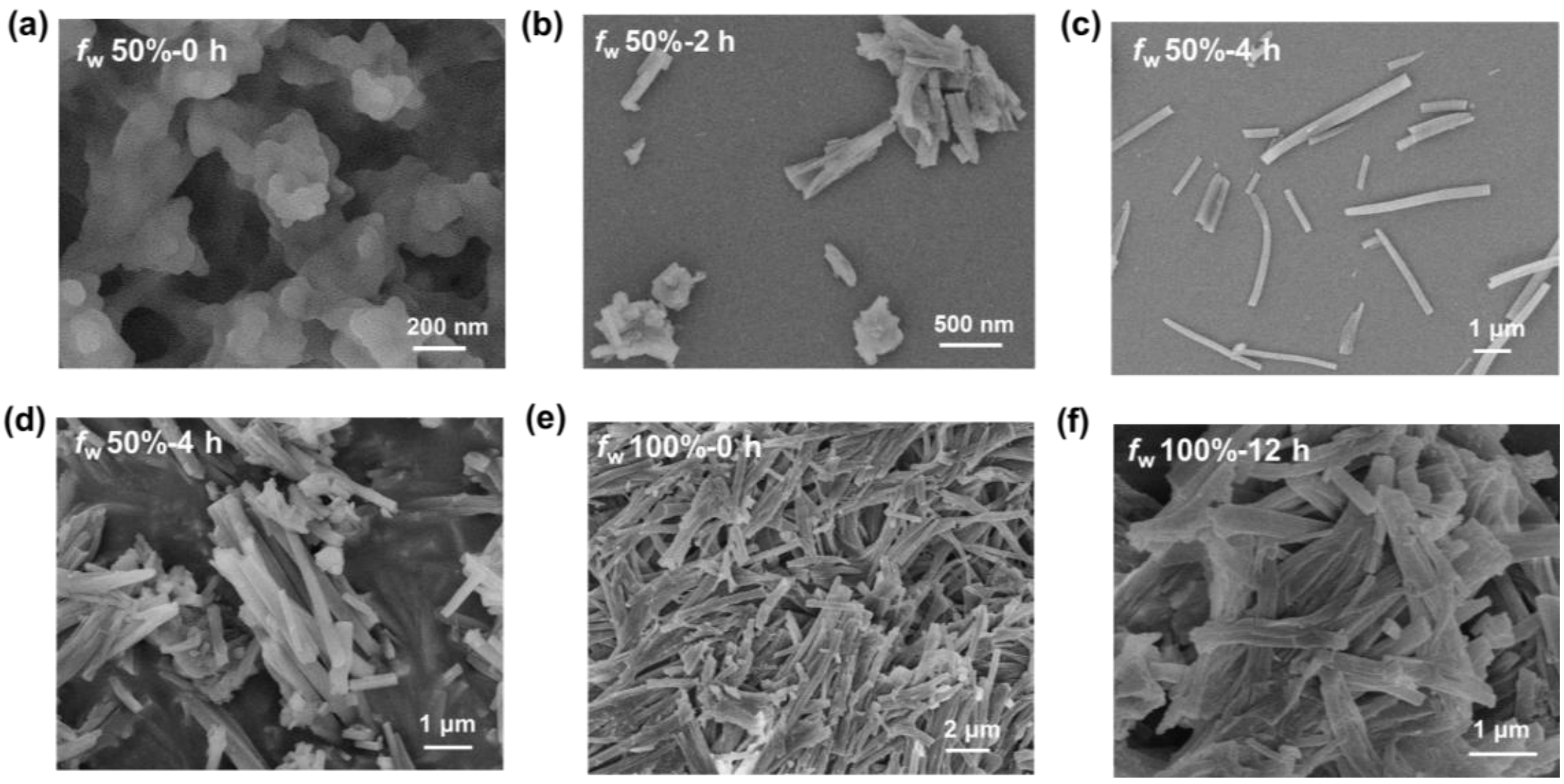

Fig. S14 (a) SEM image of freshly prepared **TCSE** at f_w = 50%. (b) SEM image of **TCSE** from a sample with 50% water fraction after standing for 2 h. (c, d) SEM images of **TCSE** from a sample with 50% water fraction after standing for 4 h. (e) SEM image of freshly prepared **TCSE** at f_w = 100%. (f) SEM image of **TCSE** from a sample with 100% water fraction after standing for 12 h.

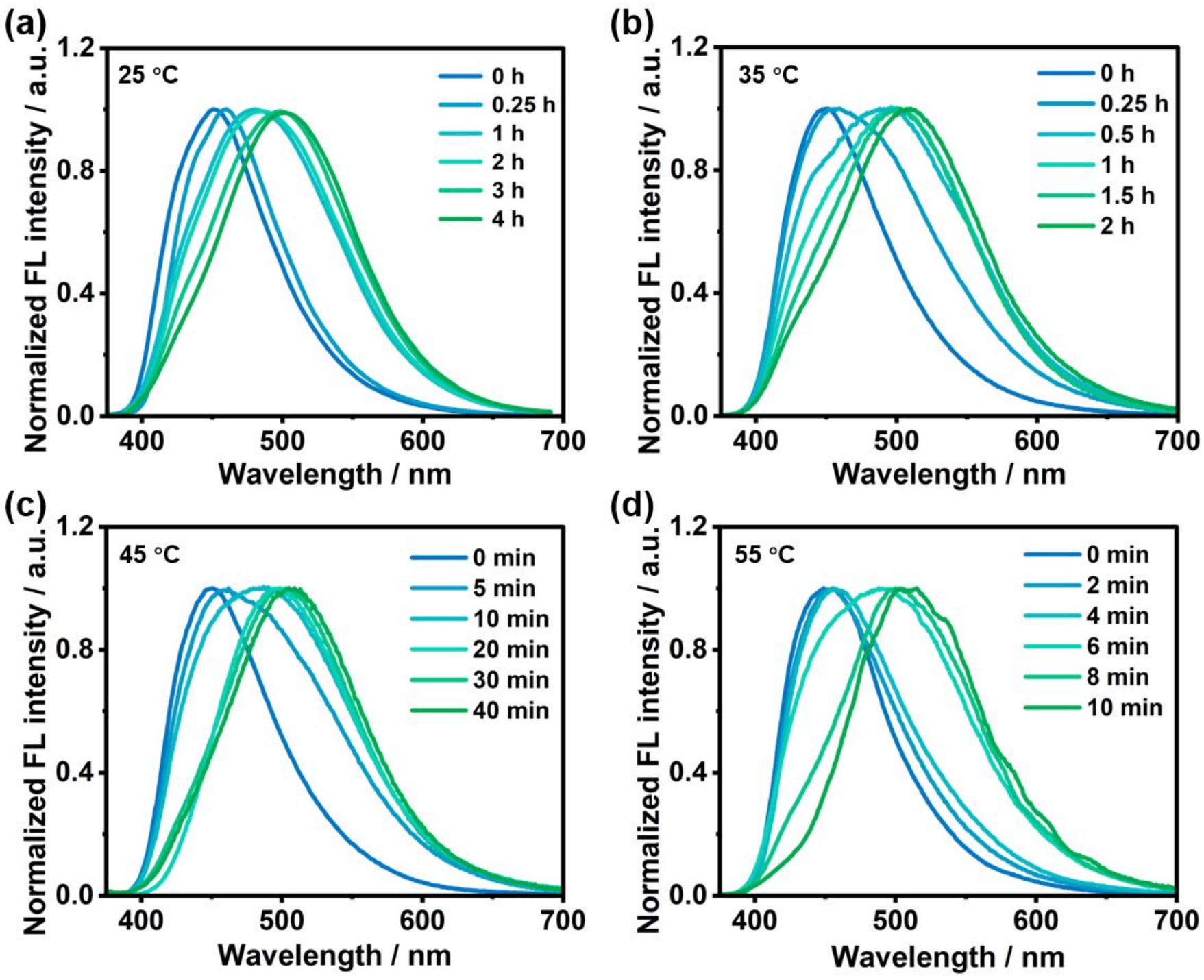

Fig. S15 Normalized fluorescence spectra of **TCSE** (f_w = 50%) at different temperatures over time: (a) 25 °C, (b) 35 °C, (c) 45 °C, (d) 55 °C. [**TCSE**] = 1.0 × 10^{-5} M, λ_{ex} = 365 nm.

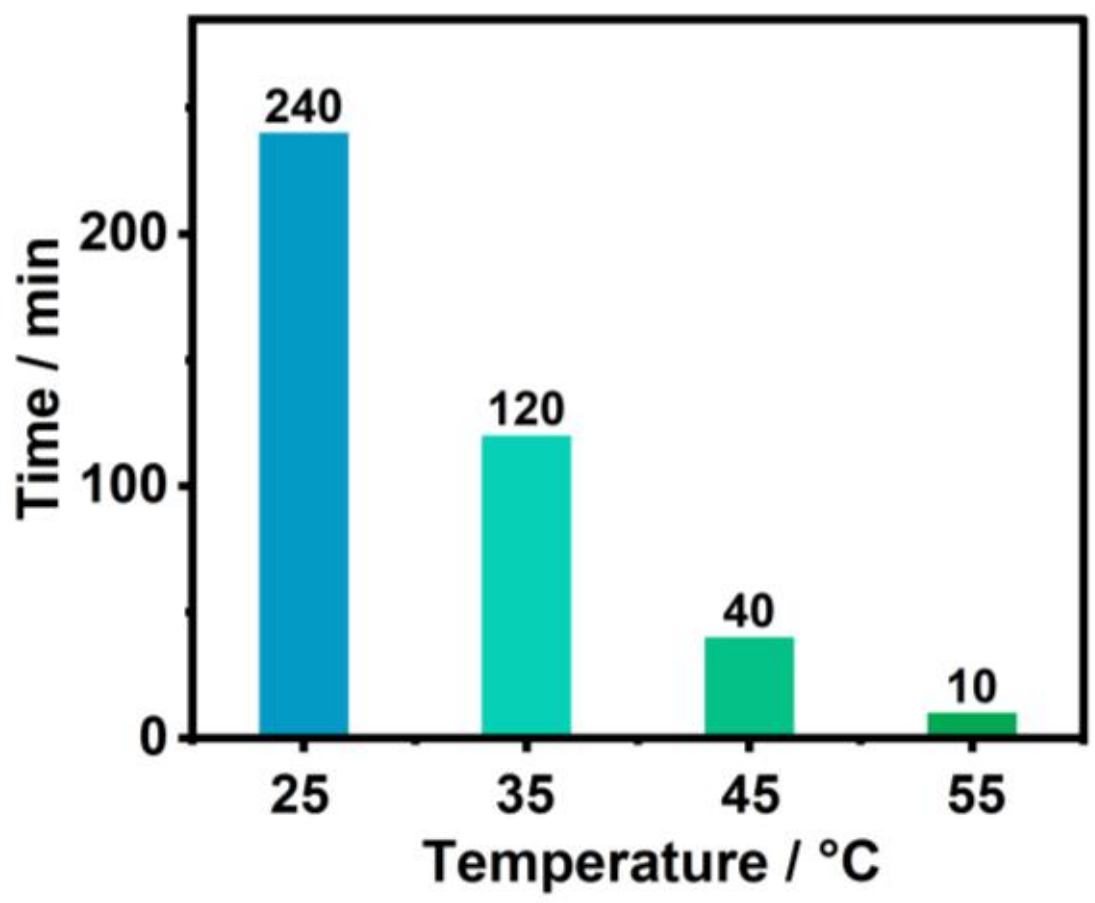

Fig. S16 Bar chart showing the temperature-dependent self-assembly time of **TCSE** (f_w = 50%) for the blue-to-green fluorescence transition. [**TCSE**] = 1.0 × 10^{-5} M.

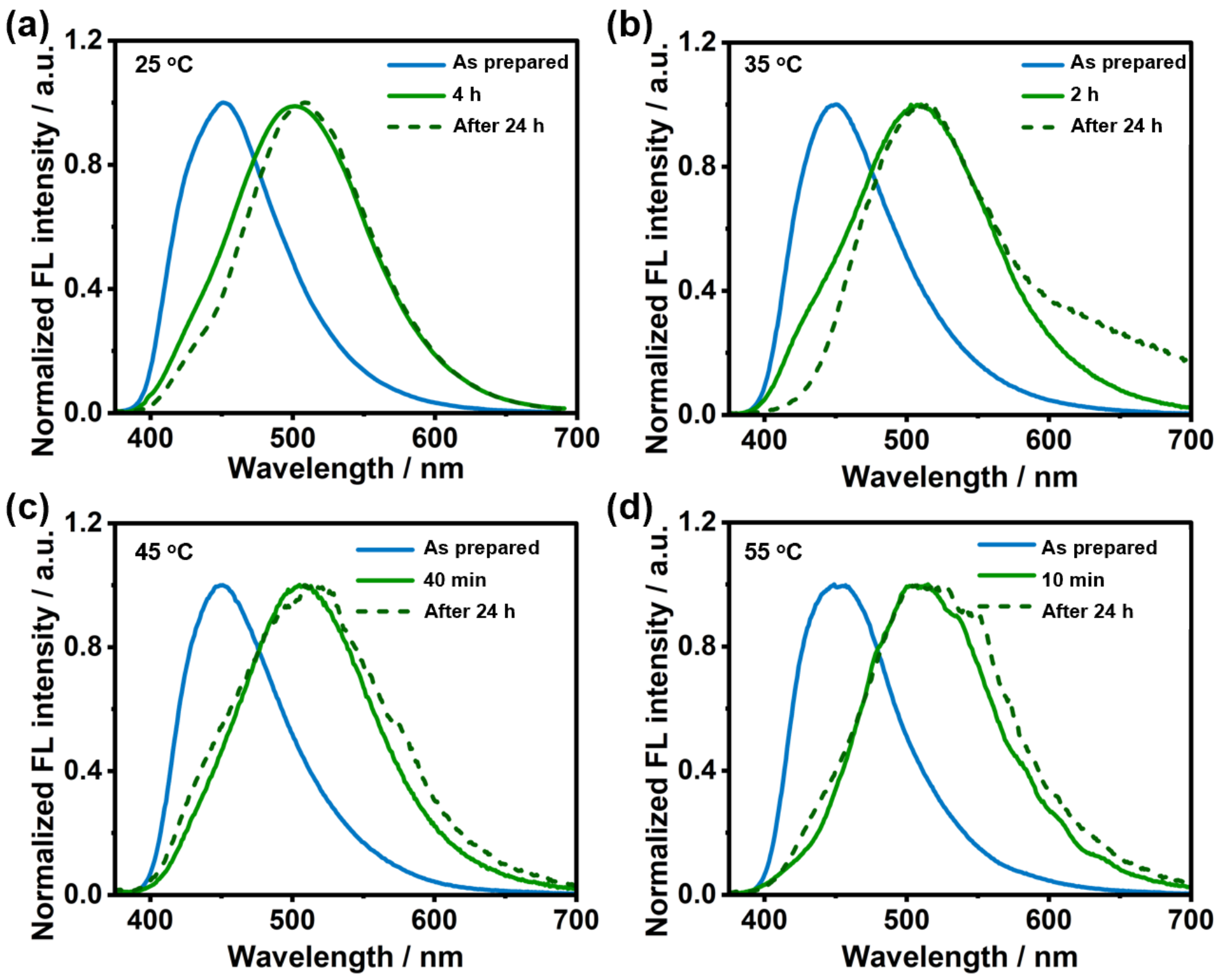

Fig. S17 Normalized fluorescence spectra of **TCSE** (f_w = 50%) before and after the time-dependent fluorescence color transition at different temperatures: (a) 25 °C, (b) 35 °C, (c) 45 °C, and (d) 55 °C. The dashed curves represent the spectra recorded after the color-changed samples were stored at room temperature for 24 h. [**TCSE**] = 1.0 × 10^{-5} M, λ_{ex} = 365 nm.

5. Information encryption based on binary code

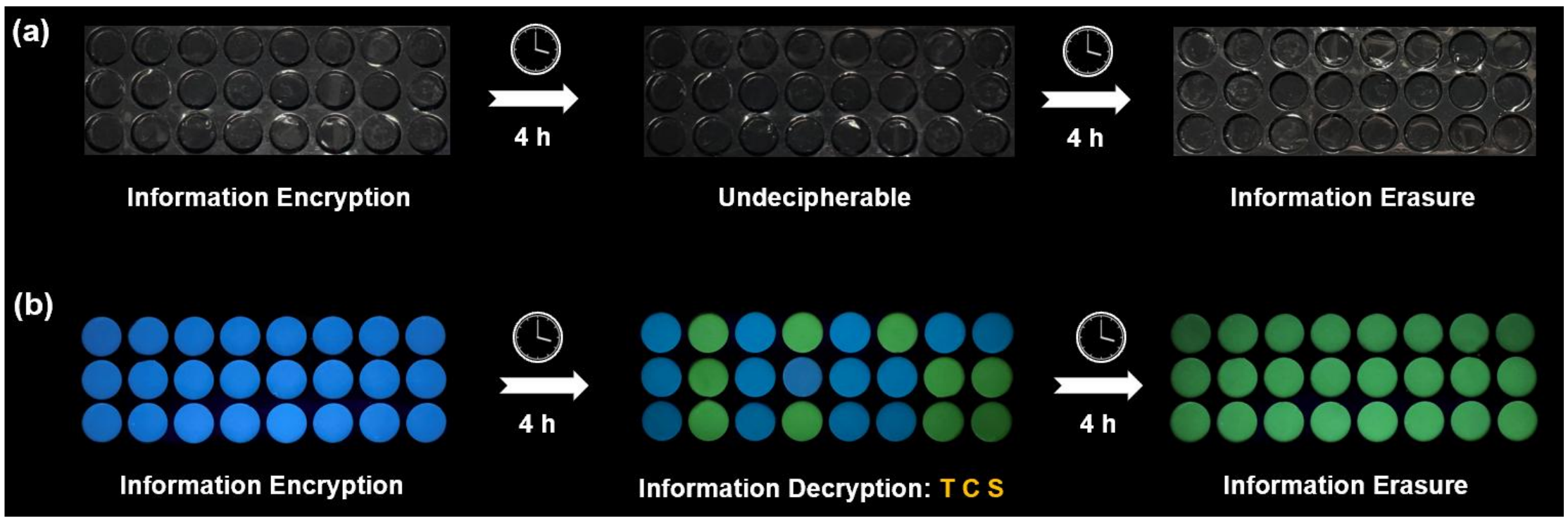

Fig. S18 (a) Photographs under daylight and (b) fluorescence images, demonstrating binary coding for anticounterfeiting constructed from **TCSE** samples with water fractions of 50% (rapid transition) and 45% (slow transition). [**TCSE**] = 1.0 × 10^{-5} M.

6. Self-erasable writing materials

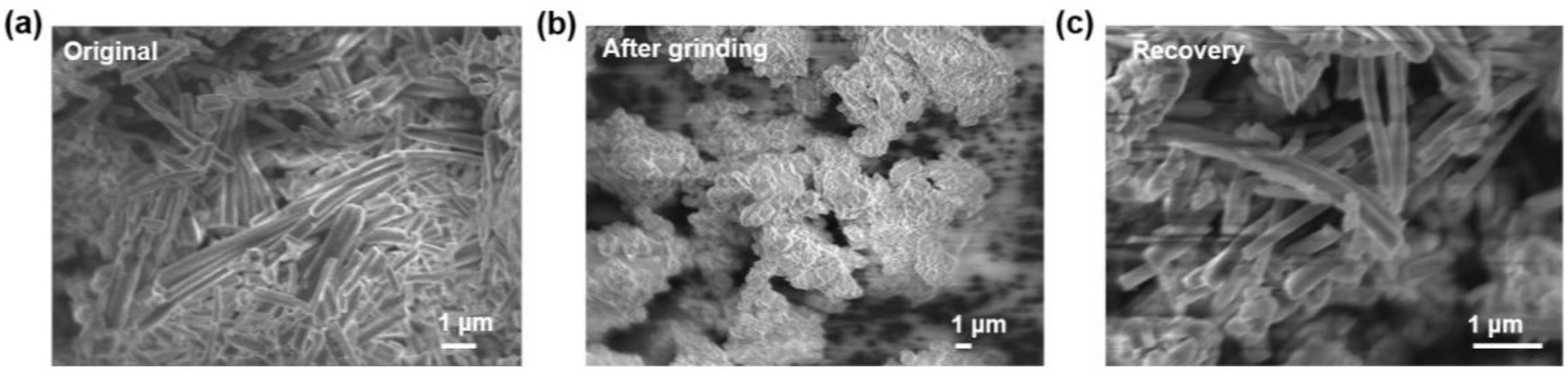

Fig. S19 SEM image of **TCSE** powder: (a) original, (b) after grinding, and (c) recovery.

Table S4 Fluorescence lifetimes and quantum yields of **TCSE** powder.

Sample	Original	After grinding	Recovery
τ/ns	13.2958	10.8851	13.0066
$\Phi_{f(abs)}$	23.04%	28.53%	23.45%

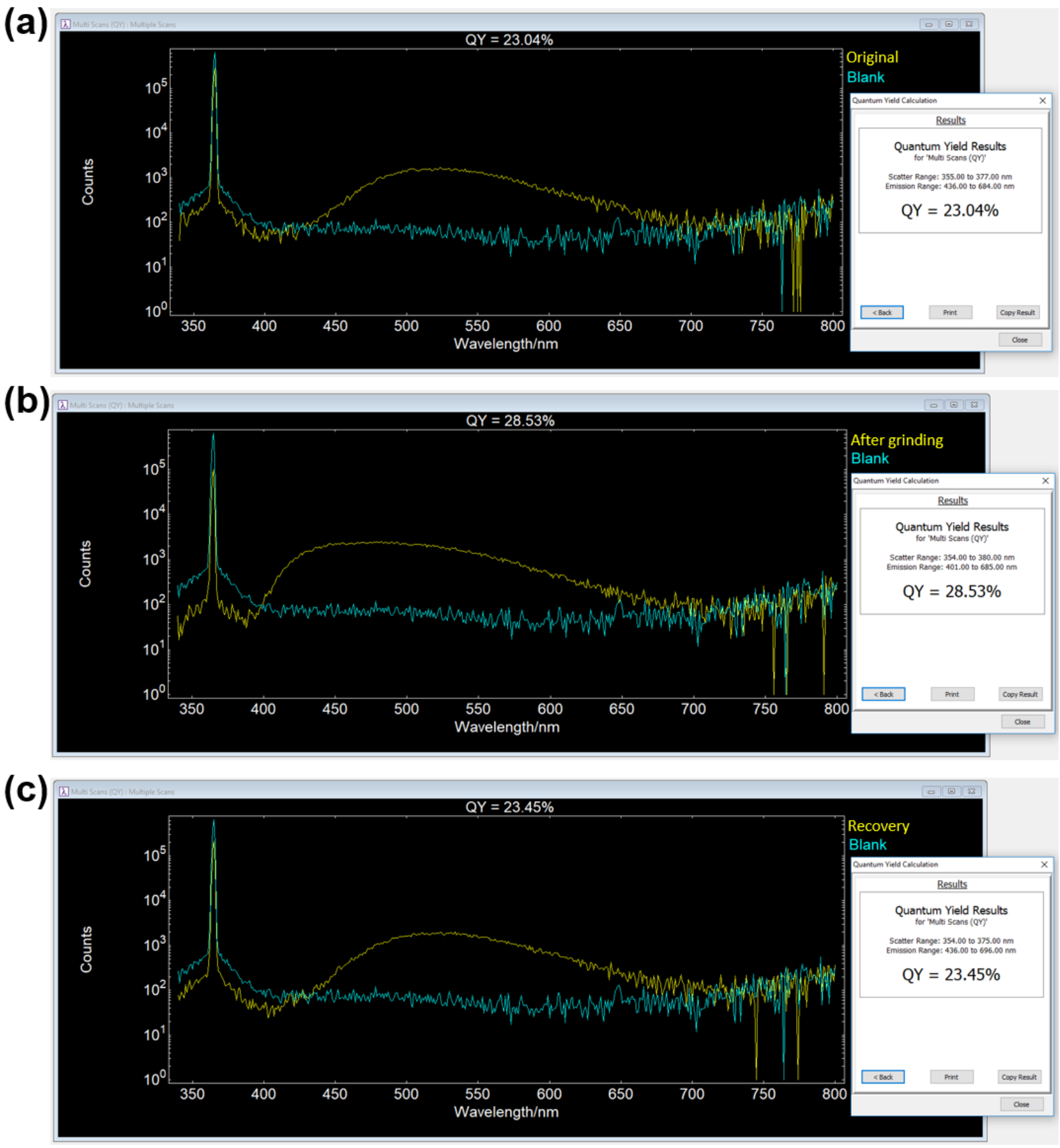

Fig. S20 Absolute fluorescence quantum yields ($\Phi_{f(abs)}$) of **TCSE** powder: (a) original, (b) after grinding, and (c) recovery upon excitation at 365 nm.

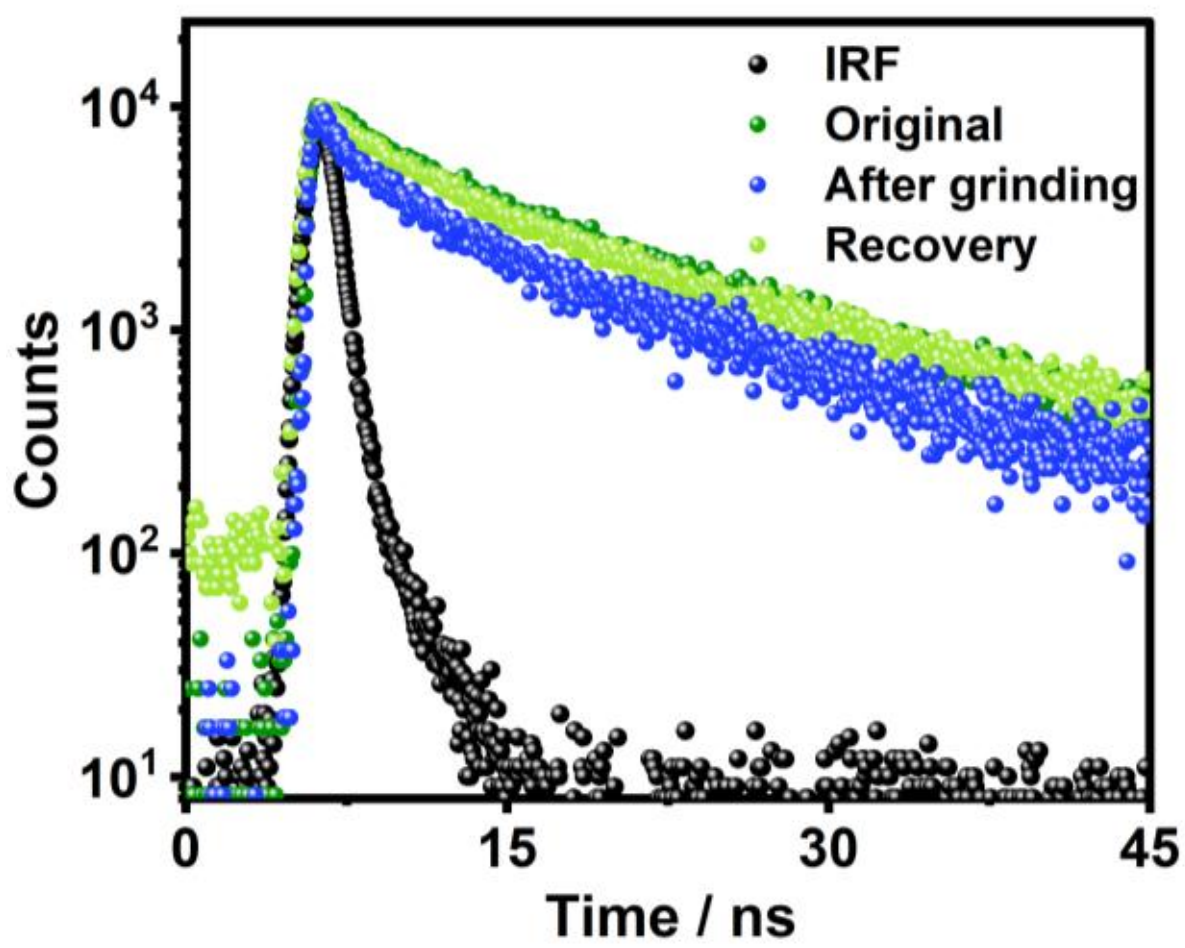

Fig. S21 Fluorescence decay profiles of **TCSE** powder: (a) original, (b) after grinding, and (c) recovery upon excitation at 365 nm.

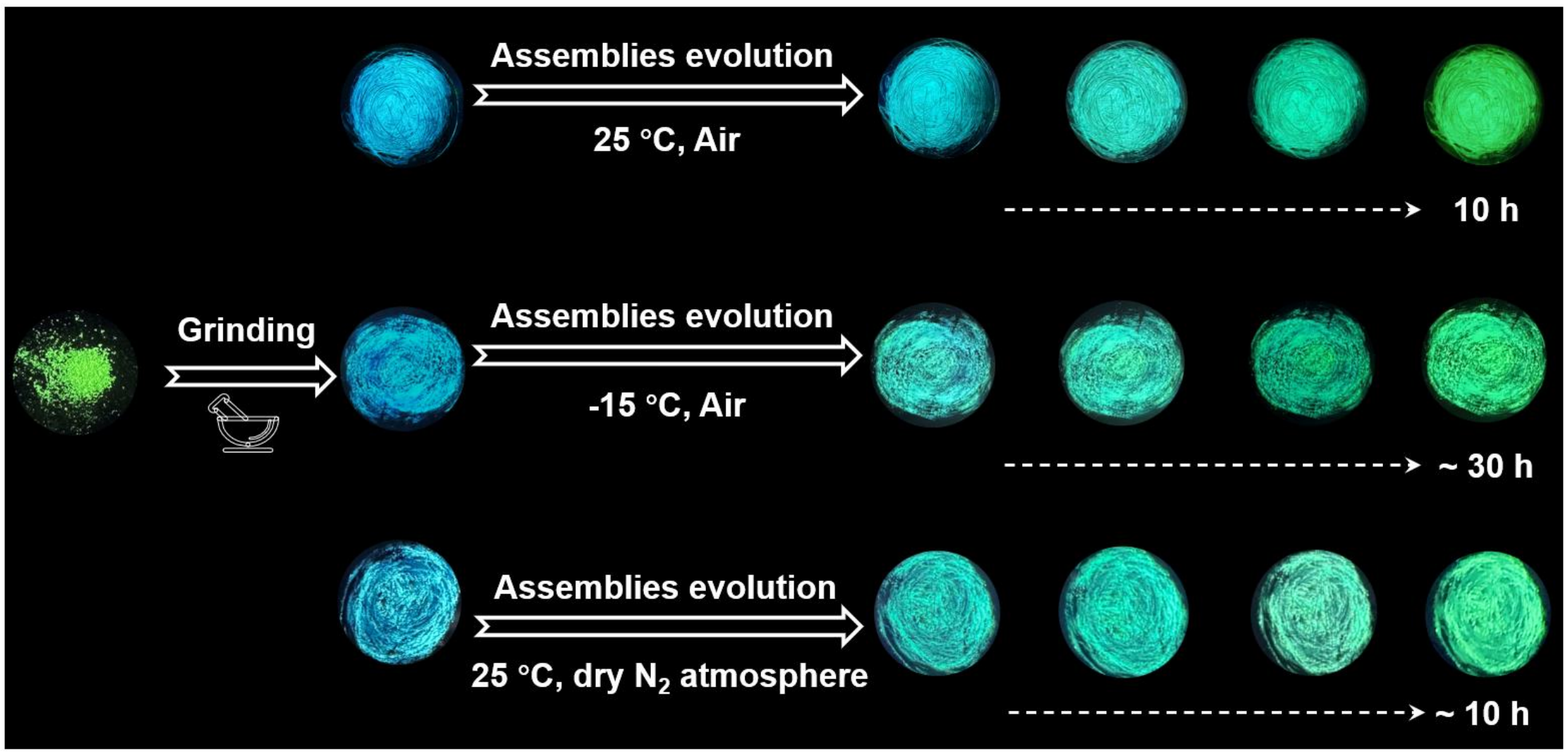

Fig. S22 Fluorescence photographs of **TCSE** powder at low temperature (-15 °C) and under dry nitrogen atmosphere.

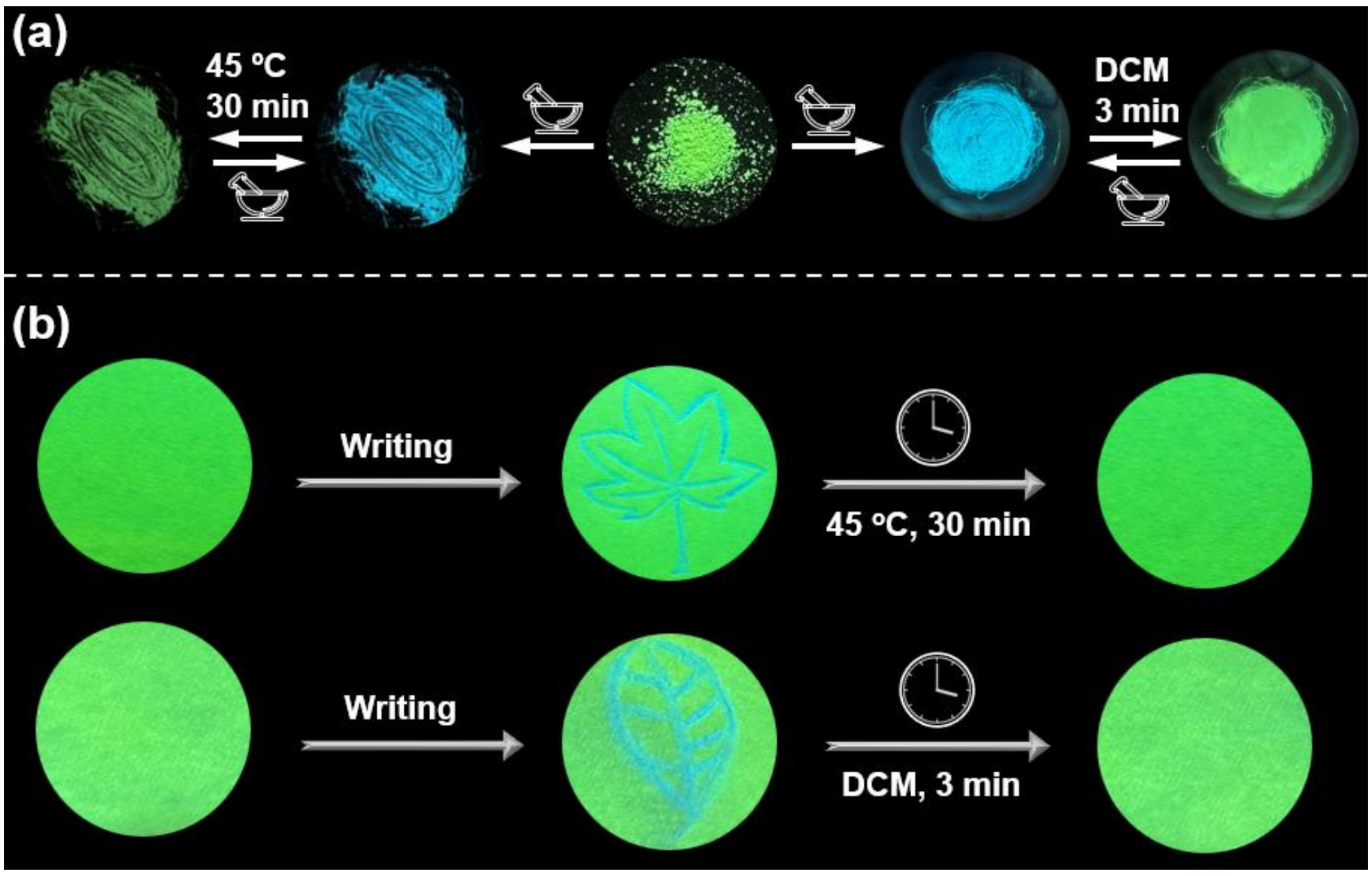

Fig. S23 (a) Fluorescence photographs of **TCSE** powder exhibiting mechanochromism and responses to heating/fuming. (b) Reversible "writing" and "erasing" demonstrated on **TCSE**-impregnated paper.

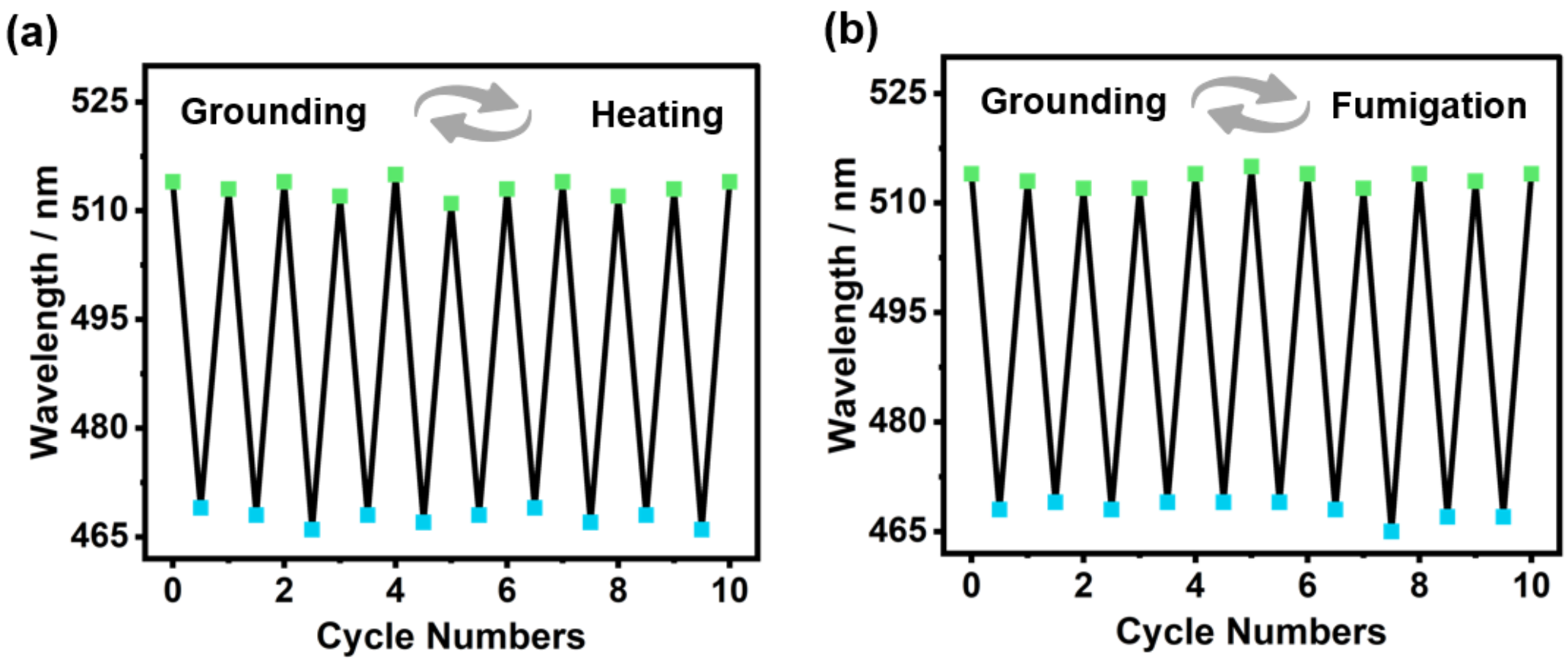

Fig. S24 Plot showing reversible switching in fluorescence emission color during cycling between grinding and recovery by (a) heating at 45 °C and (b) solvent fuming with DCM.

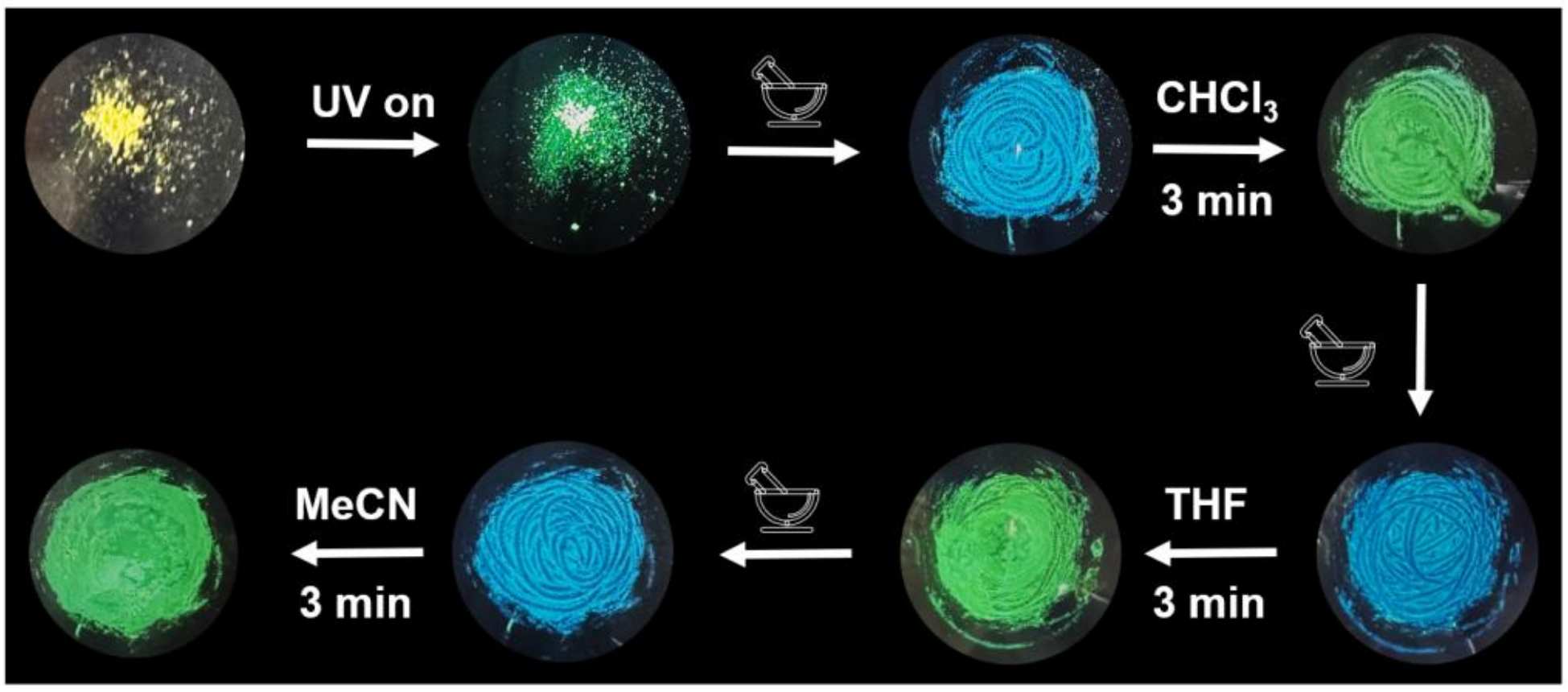

Fig. S25 Fluorescence photographs of **TCSE** powder after fuming with different solvents.

7. Comparison of TCSE with other CS systems

Table S5 Comparison of photophysical properties and dynamic behaviors among various reported cyanostilbene (CS)-based systems and **TCSE**.

System	Dynamic emission evolution	External stimulus required	Single-component system	Mechanochromism / self-erasing writing	Ref.
1 CS fluorophores	blue → yellow → white → blue evolution	Yes (Tris/GdL pH clock trigger)	No (requires myristic acid as auxiliary assembly component)	No	R[4]
	blue → white → yellow evolution	Yes (NaOH + esters trigger)	No (requires NaOH and esters as chemical triggers)	No	R[5]
	cyan → blue-cyan → yellow evolution; cyan → yellow evolution; yellow → white → blue evolution	Yes (pH + Ag^{+} + UV)	No (needs multiple chemical triggers)	No	R[6]
2 CS fluorophores	orange → green evolution	No	Yes	No	R[7]
	blue → quenched evolution	Yes (UV or TFA)	No（needs TFA as external chemical trigger）	No	R[8]
	green-yellow → blue evolution	Yes (UV + WP5)	No (needs WP5)	No	R[9]
	yellow → blue → fade evolution	Yes (UV)	Yes	No	R[10]
3 CS fluorophores (**TCSE**)	blue → green evolution	No	Yes	Yes	This work

8. References

1. B. Mu, X. Hao, X. Luo, Z. Yang, H. Lu and W. Tian, *Nat. Commun.*, **2024**, *15*, 903.
2. J.-H. Zhang, S.-M. Xie, L. Chen, B.-J. Wang, P.-G. He and L.-M. Yuan, *Anal. Chem.*, **2015**, *87*, 7817-7824.
3. M. Zhang, W. Lu, J.-R. Li, M. Bosch, Y.-P. Chen, T.-F. Liu, Y. Liu and H.-C. Zhou, *Inorg. Chem. Front.*, **2014**, *1*, 159-162.
4. P. Das, T. Das, S. Koley, M. Kumar Baroi, S. Das, J. Mohanty and D. Das, *Angew. Chem. Int. Ed.*, **2025**, *64*, e202414239.
5. Z. Zou, J. Wang, Y. Sun, Q. Wang and D.-H. Qu, *Chin. Chem. Lett.*, **2024**, *35*, 108972.
6. N. Kumar, D. Maity, S. Som and S. Banerjee, *Small*, **2025**, *21*, 2503123.
7. D. Chen, C. Bao, L. Zhang, Q. Zhang, Z. Wu, Z.-Y. Li, X.-Q. Sun, L. Wang and T. Xiao, *Adv. Funct. Mater.*, **2024**, *34*, 2314093.
8. Y. Ma, M. Cametti, Z. Džolić and S. Jiang, *J. Mater. Chem. C*, **2016**, *4*, 10786-10790.
9. Y. Wang, H. Zhong, J. Yang, Y. Yao and L. Li, *Chin. Chem. Lett.*, **2023**, *34*, 108452.
10. D. Ren, L. Tang, Z. Wu, Q. Zhang, T. Xiao, R. B. P. Elmes and L. Wang, *Chin. Chem. Lett.*, **2023**, *34*, 108617.
